# Recurrent gene co-amplification on Drosophila X and Y chromosomes

**DOI:** 10.1371/journal.pgen.1008251

**Published:** 2019-07-22

**Authors:** Christopher Ellison, Doris Bachtrog

**Affiliations:** Department of Integrative Biology, University of California Berkeley, Berkeley, California, United States of America; Fred Hutchinson Cancer Research Center, UNITED STATES

## Abstract

Y chromosomes often contain amplified genes which can increase dosage of male fertility genes and counteract degeneration via gene conversion. Here we identify genes with increased copy number on both X and Y chromosomes in various species of Drosophila, a pattern that has previously been associated with sex chromosome drive involving the *Slx* and *Sly* gene families in mice. We show that recurrent X/Y co-amplification appears to be an important evolutionary force that has shaped gene content evolution of sex chromosomes in Drosophila. We demonstrate that convergent acquisition and amplification of testis expressed gene families are common on Drosophila sex chromosomes, and especially on recently formed ones, and we carefully characterize one putative novel X/Y co-amplification system. We find that co-amplification of the *S-Lap1*/*GAPsec* gene pair on both the X and the Y chromosome occurred independently several times in members of the *D*. *obscura* group, where this normally autosomal gene pair is sex-linked due to a sex chromosome—autosome fusion. We explore several evolutionary scenarios that would explain this pattern of co-amplification. Investigation of gene expression and short RNA profiles at the *S-Lap1*/*GAPsec* system suggest that, like Slx/Sly in mice, these genes may be remnants of a cryptic sex chromosome drive system, however additional transgenic experiments will be necessary to validate this model. Regardless of whether sex chromosome drive is responsible for this co-amplification, our findings suggest that recurrent gene duplications between X and Y sex chromosomes could have a widespread effect on genomic and evolutionary patterns, including the epigenetic regulation of sex chromosomes, the distribution of sex-biased genes, and the evolution of hybrid sterility.

## Author summary

Sex chromosomes are hot spots for genetic conflict, and selfish genetic elements that increase their transmission are prone to originate on sex chromosomes. Previous work has shown that genes that co-amplify on both the X and Y chromosome are involved in sex chromosome drive in mice. Here, we use bioinformatic approaches to identify co-amplified genes in 26 Drosophila species, and we characterize a novel co-amplified gene family in *D*. *pseudoobscura* using functional genomic approaches. Our comparative genomic analysis suggests that co-amplification of genes on sex chromosomes is rampant, and we detect co-amplified X/Y genes in dozens of fly species investigated, especially those with young sex chromosomes. We find that co-amplified genes are often derived from well-characterized meiosis genes that are necessary for proper segregation, and are highly expressed in testis. Finally, we show that co-amplified genes often produce antisense transcripts and are targeted by small RNAs. Functional enrichment and expression patterns are consistent with a model where co-amplification of these genes is driven by intragenomic conflicts over transmission of the X and Y chromosome, and that RNAi mechanisms are utilized to launch evolutionary responses to counter sex ratio distortion. This would imply a novel role for the RNAi pathway to defend the genome against selfish elements that try to manipulate fair transmission.

## Introduction

Selfish genetic elements whose evolutionary trajectories are in conflict with those of their host were first described almost 100 years ago [1]. However, only in recent decades has it become apparent that the antagonistic coevolution resulting from genetic conflict has shaped genome content and structure across the tree of life, from bacteria to plants and animals [2]. Antagonistic coevolution can occur between organisms, as in the evolutionary “arms race” experienced between pathogens and their hosts, or within genomes among genetic elements with different inheritance patterns (such as mobile elements, or X and Y chromosomes [3]). For instance, selfish genetic elements can manipulate meiosis (or gametogenesis) so that they are transmitted to more than 50% of offspring (so called “segregation distorters” or “meiotic drivers”)[4]. These processes can leave behind a variety of distinct genetic signatures. For example, genes involved in pathogen virulence and host resistance consistently show elevated levels of amino acid substitutions (i.e. dN/dS) whereas genes involved in intra-genomic conflict, such as segregation distorters and their suppressors, often have high rates of lineage-specific duplications and gene amplifications [5,6]. A well-studied cryptic sex chromosome drive system in mouse involves the convergent acquisition and amplification of the same gene families (*Slx*/*Sly*) on both the X and Y chromosome, and careful experimentation has shown that the co-amplified genes are in a co-evolutionary battle over sex chromosome transmission, whereby the X-and Y-linked copies of a gene family directly compete with each other [7,8]. *Sly* knockdowns show female-biased sex ratios, while *Slx* deficiency causes a sex ratio distortion towards males. A similar mechanisms of cryptic segregation distortion has been implicated in the *Stellate*/ *Suppressor of Stellate* (*Ste*/*Su*(*Ste*)) system in *D*. *melanogaster*, where the expression of the X-linked gene *Ste* leads to the production of defective sperm, and *Su*(*Ste*), which is a multi-gene copy of *Ste* that moved to the Y-chromosome, silences *Ste* [9,10].

Although rapid rates of amino acid substitution, gene duplication, and gene amplification are all characteristics of evolutionary conflict, these processes are also associated with strong selection in the absence of conflict, or in some cases, even neutral evolution. This makes identification of conflict from genomic data alone difficult [11]. For example, recent studies have shown that the Y chromosomes of many organisms contain testes-specific genes that have amplified in copy number [12–14]. Some of these Y-linked gene families, such as those in mice, have been shown to be involved in sex chromosome drive, whereas for other gene families, the extra copies may either act to increase gene dosage or prevent degeneration by providing a substrate for non-allelic gene conversion [15]. Alternatively, the extra copies may be neutral or even slightly deleterious, yet they remain on the Y due to the reduced efficiency of selection on this non-recombining chromosome [16]. One key signature that appears to be unique to Y-amplified genes involved in sex chromosome drive is that their X-linked homologs have duplicated as well. This pattern is consistent with antagonistic co-evolution resulting from repeated bouts of sex ratio distortion and suppression (see Discussion).

Here, we use bioinformatics and functional genomic analyses to assess the prevalence of sex chromosome gene amplification across Drosophila species. Consistent with a role for Y-amplified genes unrelated to genetic conflict (see Discussion), we find hundreds of genes that appear to be present in multiple copies on the Y chromosomes of many Drosophila species. However, we also find a second category of Y-amplified genes whose X homolog has been duplicated as well. We show that species with young sex chromosomes have repeatedly evolved genes that have co-amplified on the X and the Y and show functions and expression patterns that are consistent with genetic conflict. We explore a variety of evolutionary scenarios that could give rise to this pattern of X-Y co-amplification based on detailed investigation of the *S-Lap* and *GAPsec* genes that have been independently co-amplified on the X and Y chromosomes of multiple species in the *obscura* group. We find that gene expression levels and small RNA production from these co-amplified genes are most consistent with a cryptic sex ratio drive system, however additional experiments are necessary to test these claims. We develop a model for how such a system could have evolved and present evidence suggesting that the same genes appear to have become involved in a meiotic conflict independently among multiple species of this group.

## Results

### Bioinformatic inference of co-amplified X and Y genes across Drosophila

To analyze gene content evolution and identify amplified X- and Y-linked genes, we sequenced both male and female genomic DNA in 26 Drosophila species from across the Drosophila phylogeny (S1 Table). Roughly half of the species considered (11 out of 26) harbor the typical sex chromosome complement of Drosophila (that is, a single pair of ancient sex chromosomes, shared by all members of Drosophila). In addition to the ancestral pair of sex chromosomes, the other 15 species have a younger pair of “neo-sex” chromosomes, which formed when an autosome became fused to one or both of the ancient X and Y chromosomes (S1 Fig). These younger “neo-sex” chromosomes are at various stages of evolving the typical properties of ancestral sex chromosomes, with neo-Y chromosomes losing their original genes and acquiring a genetically inert heterochromatic appearance, and neo-X chromosomes acquiring their unique gene content and sex-specific expression patterns [16,17]. We identified putative Y-amplified genes based on male and female gene coverage without relying on a genome assembly (S2 Fig, see Methods) and validated our approach using a high-quality genome assembly from *D*. *miranda* (S3 Fig). Using this approach, we identify (depending on our cutoffs) 100s of genes that have multiple copies on the Y across the 26 species investigated (S2 and S3 Tables, S4 Fig). Genes might amplify on the Y for a variety of reasons, but co-amplification of testis genes may be a defining feature of genes evolving under genetic conflict (see Discussion). Here we define co-amplified genes as being amplified on the Y, based on male and female gene coverage, and as having at least two copies on the X chromosome in our genome assemblies.

**Fig 1:**
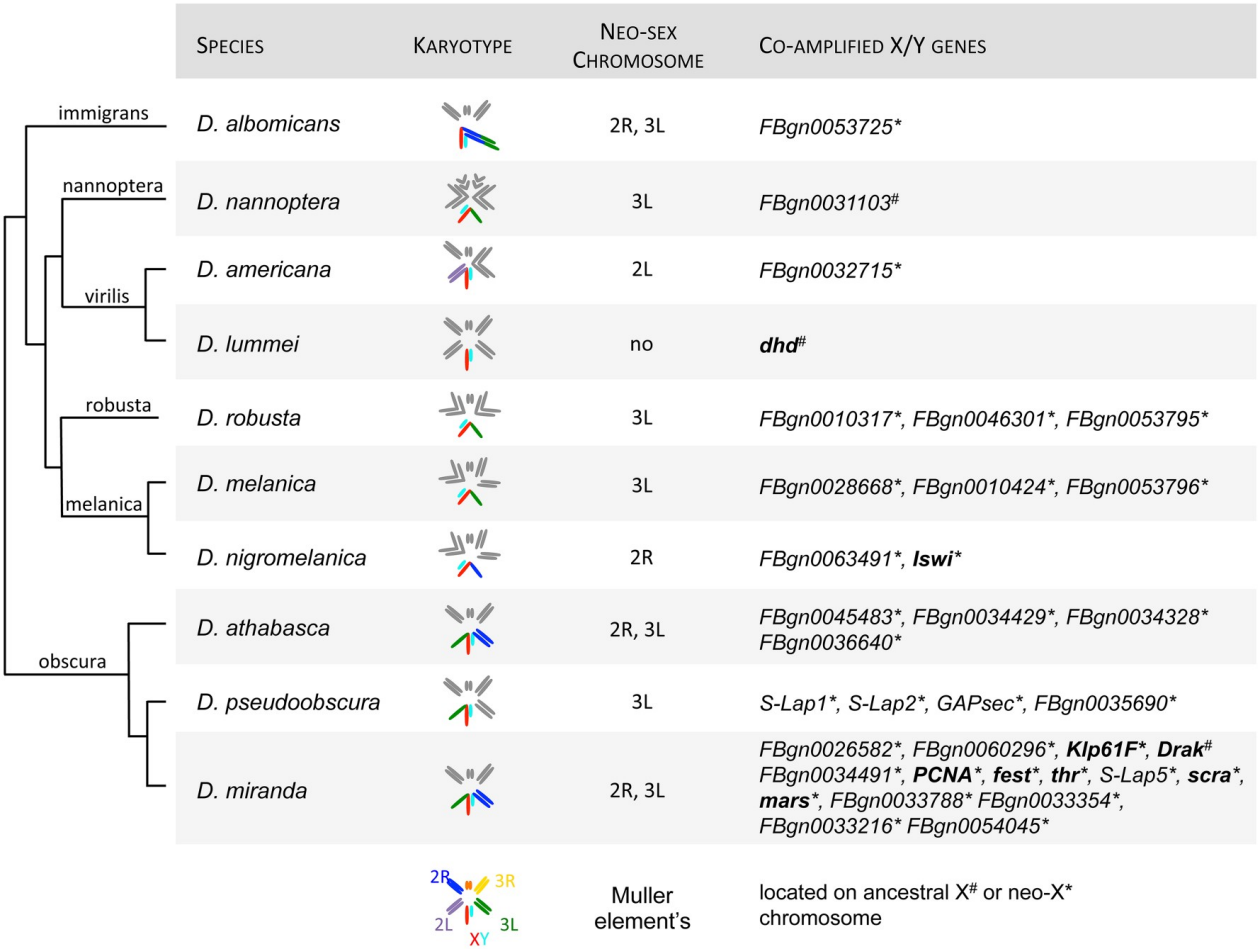
Co-amplified X and Y-linked genes across Drosophila species. Shown is the karyotype for each species with co-amplified X and Y genes, which chromosome arms form the neo-sex chromosomes (based on synteny in *D*. *melanogaster*), and the name of co-amplified X and Y genes (based on their orthologs in *D*. *melanogaster*). Genes in bold have functions related to chromosome segregation. Phylogenetic relationships are from ref. [69], and the species group is indicated above the branches.

Among these multi-copy gene families on the Y, we found 35 amplified Y-linked genes with co-amplified X homologs in 10 species (Fig 1, S4 Table). We infer that the copy number of these co-amplified X/Y genes ranges from 8 copies on the Y up to 297 Y-linked copies (for an uncharacterized testis gene in *D*. *melanogaster* that amplified on the Y of *D*. *robusta*), with a mean copy number of 58 (S5 Table). We detect between 2–4 X-linked copies in our assemblies for these co-amplified X/Y genes (S5 Table). The number of assembled X copies is likely an underestimate since recent gene duplicates are typically collapsed in assemblies derived from short read sequencing data, but investigations of high-quality genome assemblies derived from long-read technologies confirm that co-amplified genes have considerably fewer copies on the X than the Y chromosome (S6 Table; see also Discussion).

We next sought to investigate the putative functions of these co-amplified genes. We found that many are expressed in reproductive tissues in *D*. *melanogaster* (S4 Table). Of the candidate genes that we find, 76% are expressed in the testes in *D*. *melanogaster* (versus 56% genome-wide, FlyAtlas data) which is significantly more than expected by chance (one-sided Fisher’s Exact Test P = 0.011). Several of the genes have meiosis-related functions (Fig 1, S4 Table). For example, we identify genes that are associated with spindle assembly involved in male meiosis (*fest*), chromosome segregation (*mars*), or male meiosis cytokinesis (*scra*), amongst others (Fig 1). Indeed, GO enrichment analysis reveals the following terms to be enriched among co-amplified X/Y genes: sperm chromatin condensation, spindle organization, cell cycle process, and mitotic spindle organization (S5A Fig, S7 Table; note that the nominal P-values are significant but not after correcting for multiple hypothesis testing). Genes only amplified on the Y chromosome, on the other hand, show GO enrichment for different categories of metabolic processes, translation, and biosynthetic processes (S5B Fig, S7 Table).

Amplified Y genes were detected in each species investigated (S2 Table). Interestingly, however, co-amplification of genes is much more common in species with recently added neo-sex chromosomes: of the 10 species where we found co-amplified genes, nine harbor neo-sex chromosomes (Fig 1), and in the vast majority of cases the amplified genes were ancestrally present on the chromosome that formed the neo-sex chromosomes (Fig 1).

### Characterization of *S-Lap1* / *GAPsec* gene family in *D*. *pseudoobscura*

**Fig 2:**
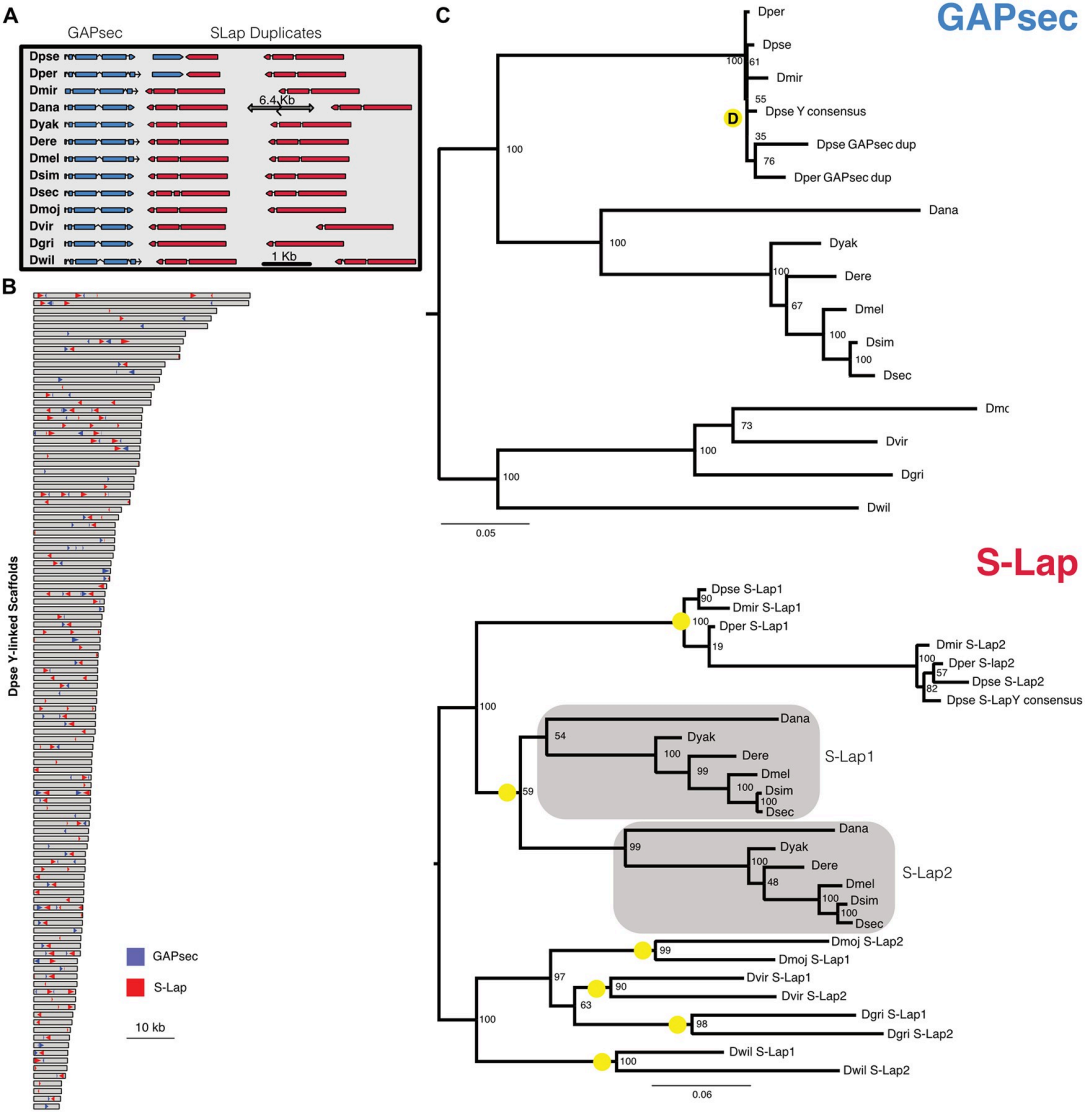
Molecular evolution of the *S-Lap1* and *GAPsec* genes in Drosophila. **A**. Organization of *S-Lap1* and *GAPsec* genes across the Drosophila genus. *S-Lap1* (shown in red) is duplicated in all Drosophila species investigated, and *GAPsec* (shown in blue) shows a partial duplication in *D*. *pseudoobscura* and its sister species *D*. *persimilis*. **B**. Amplification of *S-Lap1* and *GAPsec* on Y-linked scaffolds of *D*. *pseudoobscura*. Each rectangle represents a Y-linked genomic scaffold whose size is proportional to the scaffold length. The location and orientation of *S-Lap1* (red) and *GAPsec* (blue) duplicates on each scaffold is represented by the colored arrowheads. **C**. Gene trees of *S-Lap1* and *GAPsec* copies. A RAxML maximum likelihood phylogeny was inferred from alignments of the *GAPsec* and *S-Lap* transcripts for 12 Drosophila species plus *D*. *miranda*. The conserved genomic location of *S-Lap1* and *S-Lap2* across Drosophila suggests that they duplicated in an ancestor of Drosophila, and that gene conversion (indicated by the yellow circles) homogenized these tandem duplicates at different branches along the phylogeny. An inferred duplication event of *GAPsec* is shown by the yellow circle with a D. Alignments used to generate the trees shown in Fig 1C are provided as S1 and S2 Data.

**Fig 3:**
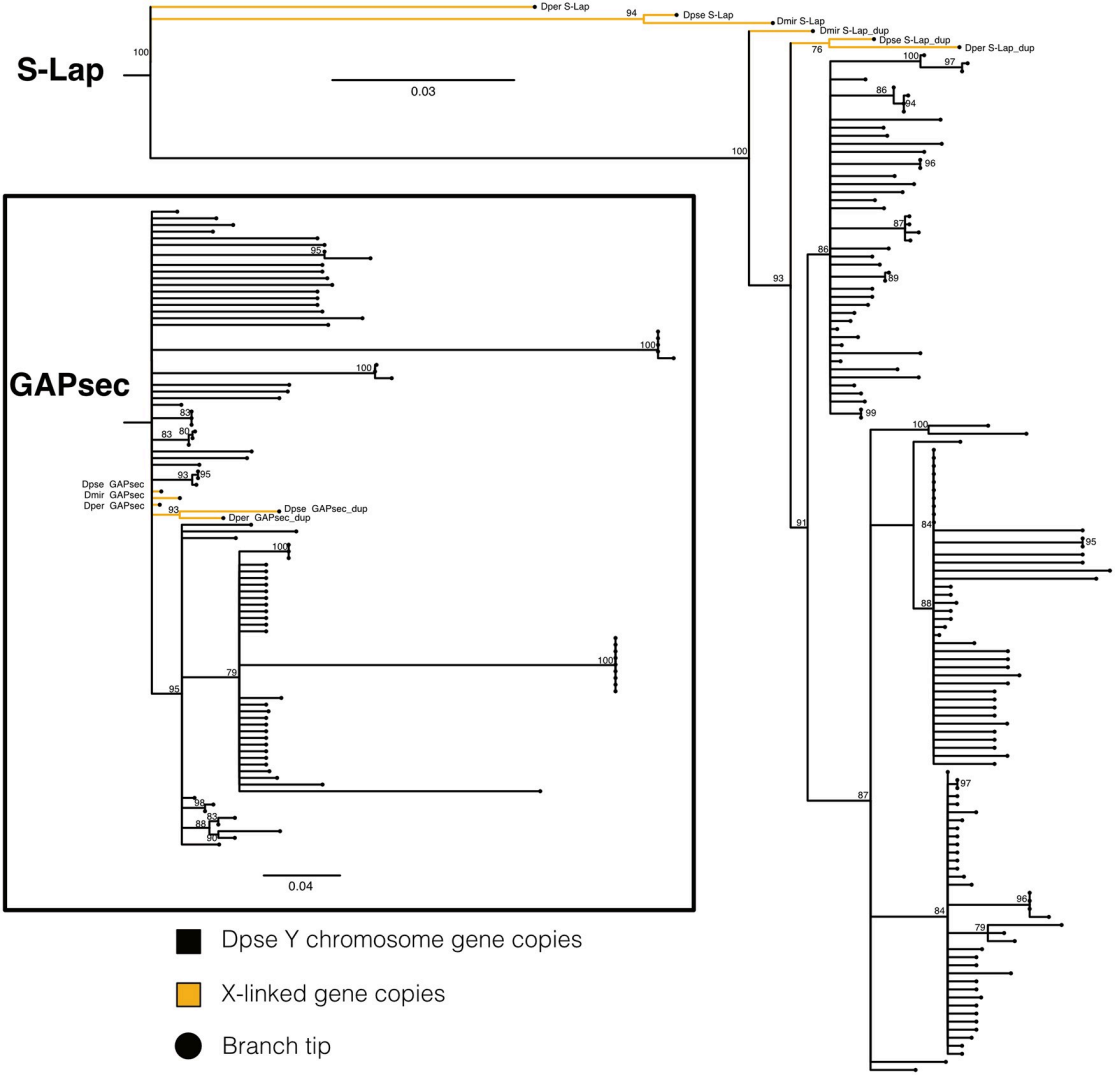
Phylogeny of *S-Lap1* and *GAPsec* gene families in *D*. *pseudoobscura*. Approximately 100 copies of both *S-Lap1* and *GAPsec* are present on *D*. *pseudoobscura* Y-linked genomic scaffolds. A RAxML maximum likelihood phylogeny was inferred from alignments of these copies and nodes with bootstrap support values of 75 or less were collapsed. X-linked copies are shown by orange branches, and Y-linked copies are shown in black. Alignments used to generate the trees shown in Fig 2 are provided as S3 and S4 Data.

**Fig 4:**
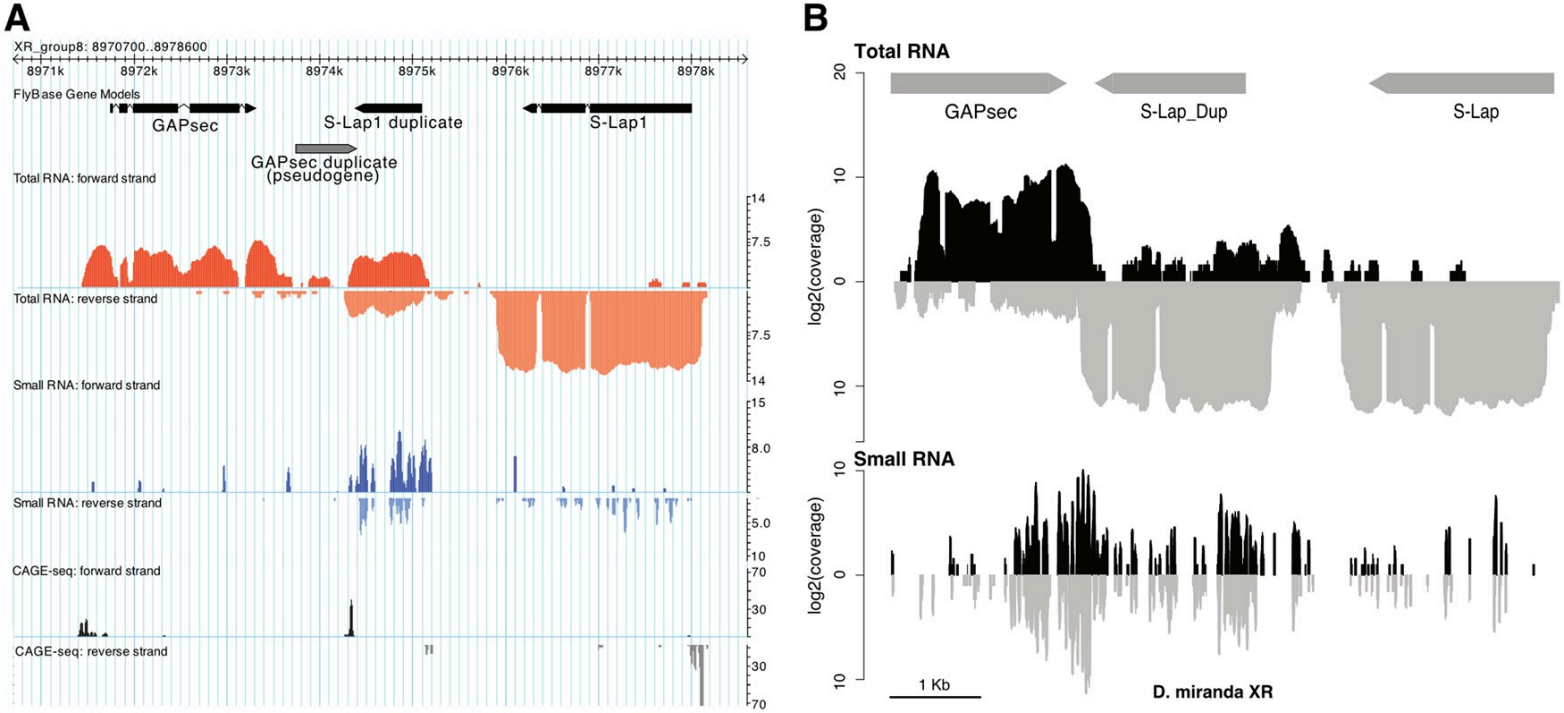
Antisense transcripts and small RNAs are produced from the *S-Lap1* and *GAPsec* gene families. **A**. Expression and small RNA profiles from wildtype *D*. *pseudoobscura* testis. Stranded RNA-seq (red tracks) reveals that the X-linked copy of *S-Lap1*-duplicate produces both sense and anti-sense transcripts, resulting in the production of small RNAs (blue tracks). CAGE-seq data (grey tracks) support that the *GAPsec* duplicate generated a new TSS resulting in antisense transcript of *S-Lap1*-dup. **B**. Expression and small RNA profiles from wildtype *D*. *miranda* testis. The *D*. *miranda* copies of both *GAPsec* and *S-Lap1*-dup produce both sense and antisense transcripts as well as small RNAs. The antisense transcripts appear to be generated from transcriptional read-through of both genes. Gene expression data are given in S5 Data.

We decided to more carefully characterize two co-amplified genes in *D*. *pseudoobscura*, a species with a high quality PacBio-based genome assembly. *D*. *pseudoobscura* currently lacks an assembled Y chromosome, but we inferred Y-linkage of contigs based on male and female read coverage using Illumina data (see Methods). We identified two adjacent genes that exist in multiple copies on the X and Y chromosome of *D*. *pseudoobscura*: *S-Lap1* (Dpse\GA19547) and *GAPsec* (Dpse\GA28668). *S-Lap1* is a member of a leucyl aminopeptidase gene family that encodes the major protein constituents of Drosophila sperm [18], while *GAPsec* is a GTPase activating protein. This situation is reminiscent of the *Segregation distorter* meiotic drive system in *D*. *melanogaster*, where the distorter is a truncated tandem duplication of RanGAP, which is also a GTPase activator [19]. Both *S-Lap1* and *GAPsec* show partial tandem duplications on the X (Fig 2A), and we detect roughly 100 (partial and full-length) copies of both *S-Lap1* and *GAPsec* on the Y chromosome (the Y-linked contigs contain 127 copies of *S-Lap1* and 91 copies of *GAPsec*; Figs 2B and 3). *S-Lap1* and *S-Lap2* are present in all Drosophila species investigated (Fig 2A), and probably originated in an ancestor of Drosophila; phylogenetic clustering of *S-Lap1* and *S-Lap2* in certain species groups (Fig 2C) probably resulted from gene conversion homogenizing gene duplicates within a clade [20]. In most species of the Drosophila clade, *S-Lap1* and *S-Lap2* are similar in size; in *D*. *pseudoobscura* and its sister *D*. *persimilis*, however, *S-Lap2* has acquired a large deletion, removing more than half of the 3’ end of the gene (Fig 2A and 2C; S6A Fig). The partial duplication of *GAPsec*, on the other hand, is only found in *D*. *pseudoobscura* and its close relative *D*. *persimilis* (Fig 2A and 2C; S6B Fig). *S-Lap1* and *GAPsec* probably dispersed onto the Y chromosome simultaneously, as there are multiple locations on the Y that preserve their X orientation (Fig 2B); note however, that the amplified copies on the Y do not include the tandemly duplicated copies. For both *S-Lap1* and *GAPsec*, the X- and Y-linked copies are highly expressed in testis of *D*. *pseudoobscura* (S4 and S8 Tables, Fig 4A).

We used stranded RNA-seq and small RNA profiles from wildtype *D*. *pseudoobscura* testes, to obtain insights into the evolutionary mechanism responsible for the co-amplification of *S-Lap1* and *GAPsec*. Interestingly, we detect both sense and antisense transcripts and small RNAs derived from *S-Lap1* (Fig 4A, S8 Table; see S7 Fig for the size distribution of small RNAs mapping and S8 Fig for cross-mapping of RNA-seq and small RNA reads between different gene copies). In particular, stranded RNA-seq data reveal that the X-linked copy of *S-Lap1* duplicate produces both sense and anti-sense transcripts, resulting in the production of small RNAs (see Fig 4A, S8 Table). Close inspection of this genomic region in *D*. *pseudoobscura* shows that the duplicated *GAPsec* gene is directly adjacent to where the *S-Lap1* duplicate antisense transcript begins (Fig 4A). Intriguingly, this segment scores highly as a potential promoter sequence when using the Berkeley Drosophila Genome Project (BDGP) neural network promoter prediction algorithm [21] (score = 0.89, highest possible score = 1). Thus, this suggests that the partial duplication of *GAPsec* provided a promoter-like sequence in *D*. *pseudoobscura* for antisense transcription of *S-Lap1* duplicate. Note that this putative promoter sequence is not part of the Y copies of *S-Lap1* (which lack the *GAPsec* duplicate), and we detect virtually no antisense transcripts that originate from the Y-amplified copies of *S-Lap1* (S8 Table). CAGE-seq data support that the *GAPsec* duplicate generated a new TSS resulting in antisense transcription of *S-Lap1* duplicate (Fig 4A).

How unusual is antisense RNA expression, and the production of small RNAs for testis genes? To see if these features of *S-Lap1* are commonly observed for other genes in *D*. *pseudoobscura* testis, we used our RNA-seq and small RNA data to identify additional genes that are expressed in testis (rpkm> = 2), show antisense expression (at least 75% of sense expression), and the production of small RNAs (rpkm> = 100). In addition to the *S-Lap1* gene, this screen revealed 6 additional genes that produced antisense transcripts and small RNAs in the testes of *D*. *pseudoobscura* (S9 Fig, S9 Table). Interestingly, all of the identified genes are members of gene families (i.e. we detect at least 2 gene copies in the *D*. *pseudoobscura* assembly), and for 5 out of 6 genes, at least one copy is located on one of the sex chromosomes, and another copy is found on the other sex chromosome, or an autosome.

### Independent co-amplification of *S-Lap1* and *GAPsec* in other species of the *Drosophila obscura* group

**Fig 5:**
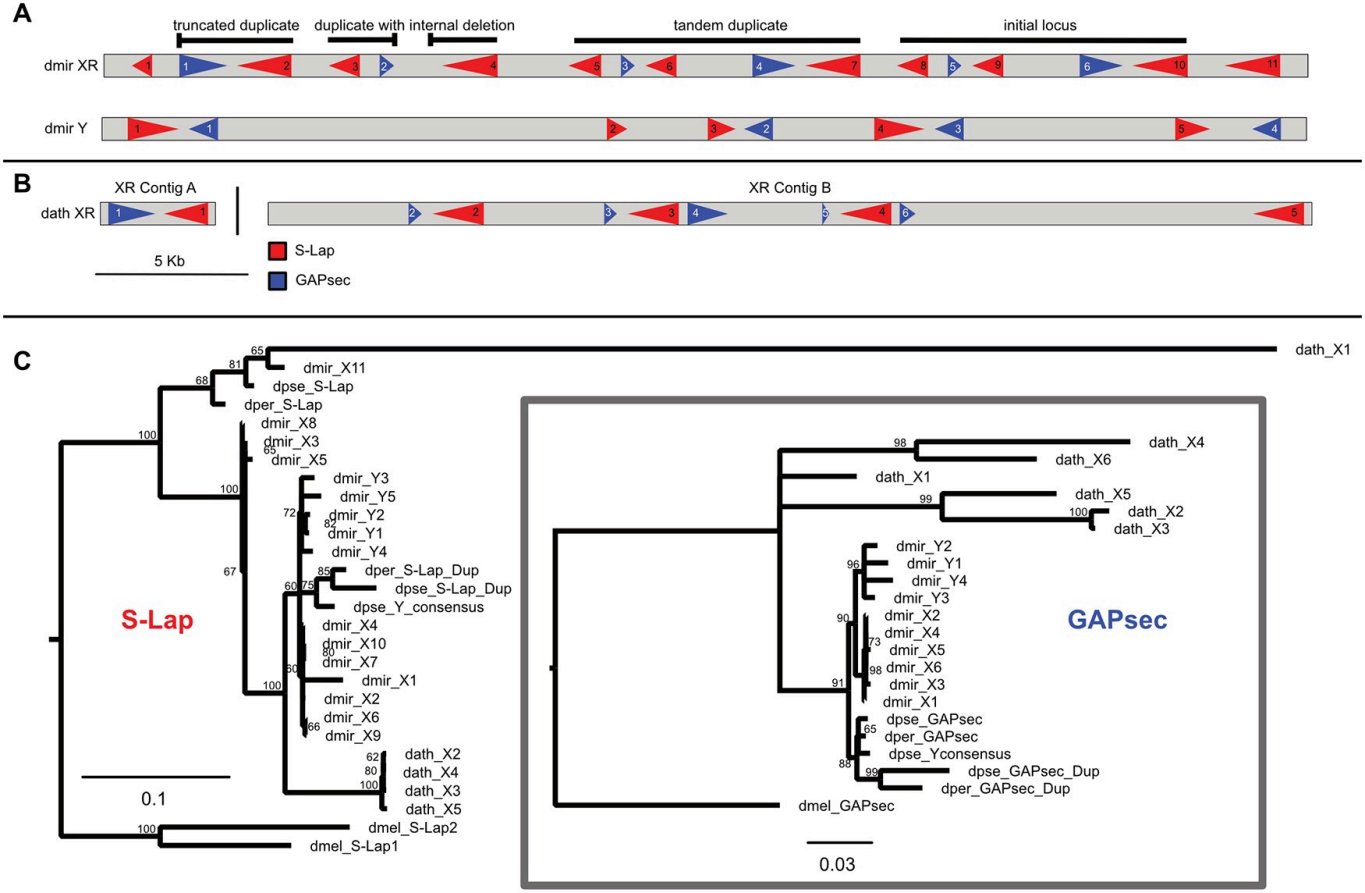
Independent amplification of *S-Lap1* / *GAPsec* in different species of the *obscura* group. **A**. Genomic organization of *S-Lap1* (red) and *GAPsec* (blue) genes on the X (dmir XR) and Y (dmir Y) chromosomes of *D*. *miranda*. We detect 11 copies of *S-Lap1/2* and 6 copies of *GAPsec* on the X chromosome, and 5 copies of *S-Lap1/2* and 4 copies of *GAPsec* on the Y chromosome of *D*. *miranda*. **B**. Genomic organization of *S-Lap1* and *GAPsec* genes on the X (dath XR) of *D*. *athabasca*. We detect 5 copies of *S-Lap1/2* and 6 copies of *GAPsec* on the X chromosome of *D*. *athabasca*. No assembly of the Y chromosome for *D*. *athabasca* exists. The location and orientation of each gene is represented by a single arrowhead. The arrowhead numbers correspond to the gene copy names in the gene trees. **C**. Gene trees of *S-Lap1* and *GAPsec* copies of *D*. *miranda* and *D*. *athabasca*. RAxML maximum likelihood phylogenies were inferred from multiple sequence alignments of the gene copies shown in (A) and (B). Nodes with bootstrap support values less than 50 are collapsed. Alignments used to generate the trees shown in Fig 4C are provided as S6 and S7 Data.

In most Drosophila species, *S-Lap1* and *GAPsec* are located on an autosome (chromosome 3L in *D*. *melanogaster*). In the *D*. *pseudoobscura* and *affinis* group, however, this chromosome arm fused with the sex chromosomes about 15MY ago, causing *S-Lap1* and *GAPsec* to become sex-linked. Intriguingly, patterns of molecular evolution at *S-Lap1* and *GAPsec* suggest that they may have independently co-amplified in several members of the *pseudoobscura* species group. We used high-quality PacBio genome assemblies for two additional members of that species group [22], *D*. *miranda*, which diverged form *D*. *pseudoobscura* about 2–4 MY ago, and *D*. *athabasca*, which diverged 10–15 MY ago [23]. While our Illumina sequencing-based approach failed to detect co-amplified X and Y genes in these species (they have similar M/F coverage), examination of the assembled PacBio genomes revealed that both gene pairs independently amplified on the sex chromosomes of both *D*. *miranda* and *D*. *athabasca* (see Fig 5). We identify tandem duplications of the entire genomic region containing a total of 11 copies of *S-Lap1* and 6 copies of *GAPsec* on chromosome XR in *D*. *miranda*, and these two genes have amplified 5 and 4 times, respectively, on the neo-Y chromosome of *D*. *miranda* (Fig 5A). Both the nature of the duplication event and patterns of sequence evolution suggest that co-amplification of *S-Lap1* and *GAPsec* occurred independently in *D*. *miranda*. Here, the XR copies arose from individual duplications of these two genes followed by three tandem duplications of the entire genomic region encompassing *S-Lap1* and *GAPsec*, producing a total of 11 copies of *S-Lap1* and 6 copies of *GAPsec*. All six of the X-linked copies of *GAPsec* are highly similar to each other (>99% identical), and more similar to their Y-linked paralogs than they are to *D*. *pseudoobscura* (Fig 5C). Also, *S-Lap1* and *GAPsec* appear to have moved only to a single location on the neo-Y of *D*. *miranda*, instead of being dispersed all across the Y, as in *D*. *pseudoobscura* (Fig 5A). Patterns of gene expression and short RNA production in *D*. *miranda* mimic that of *D*. *pseudoobscura*, with *SLap-1* (and *GAPsec*) transcripts being produced from both strands, and small RNAs are generated across that genomic region (Fig 4B). The mechanism of antisense production appears to differ from *D*. *pseudoobscura* (Fig 4). In particular, transcriptional read-through at both *S-Lap1* and *GAPsec* appear to generate anti-sense transcripts of both genes. Close inspection of this genomic region in *D*. *miranda* reveals sequence differences between the X and Y copies that may account for antisense production at X-linked gene copies. In particular, we detect a polyadenylation signal (AATAAA) for *GAPsec* that is present in most (3 of the 4) Y copies, and in the homologous copy on the X in *D*. *pseudoobscura*, but which is missing in the *D*. *miranda* X-linked copies of *GAPsec*. This mutational event could account for the creation of read-through transcripts on the X of *D*. *miranda*, leading to production of antisense transcripts for *S-Lap1* and initiation of RNAi, analogous to the model proposed for *D*. *pseudoobscura*.

Likewise, we infer independent amplifications of *S-Lap1* and *GAPsec* on the X chromosome of *D*. *athabasca*, another member of the *pseudoobscura* group for which we have generated a high-quality female genome assembly (Fig 5B). We detect 6 copies of *GAPsec* and 5 copies of *S-Lap1* on the X chromosome of *D*. *athabasca*, and genomic coverage analysis suggests a similar number of copies on the Y chromosome (i.e. males show similar coverage of *S-Lap1* and *GAPsec* as females, suggesting similar copy numbers on the X and Y). This suggests that *S-Lap1* and *GAPsec* are independently involved in co-amplification in species where this locus has become sex-linked.

## Discussion

Our comparative analysis shows that co-amplification of genes on sex chromosomes is common in Drosophila. Note, however, that our method for identifying co-amplified X/Y genes is conservative, and we might greatly underestimate the true magnitude of co-amplification. On one hand, our approach for detecting amplified Y-genes requires them to have much higher coverage in male than female genomic reads (i.e. 2.5-fold higher coverage), and can thus only detect genes that have acquired considerably more copies on the Y chromosome relative to the X or autosomes. Indeed, our recent careful examination of gene family evolution on the fully sequenced and assembled neo-Y of *D*. *miranda* confirms that the true number of co-amplified X/Y gene families is much higher than what we can detect here: Direct sequence inspection revealed that at least 94 genes co-amplified on the X and Y of *D*. *miranda* [24], while we could only identify 15 genes with our methodology. In addition, we only probed for genes that are present in the *D*. *melanogaster* annotation. Most of the species that we surveyed here are only distantly related to *D*. *melanogaster*, and many genes from other species may simply not have a homolog in *D*. *melanogaster*. Indeed, about 1/3 of the co-amplified X/Y genes that we identified in *D*. *miranda* did not have an ortholog in *D*. *melanogaster* [24]. Finally, we required X-linked co-amplified gene copies to be present in our Illumina assemblies; however, recent gene duplicates are often collapsed is such assemblies [22]. Thus, our current list of co-amplified X/Y genes may only be the tip of the iceberg, and careful examination of high-quality genome sequences of X and Y chromosomes in many taxa may reveal the true extent of gene (co)-amplification on sex chromosomes.

### Is co-amplification of sex-linked genes in Drosophila due to genetic conflict?

Genes may amplify on the Y chromosome for a variety of reasons, and our current data do not allow us to evaluate their relative importance. In particular, multi-copy genes may simply arise on the Y at a higher rate, since the high repeat content on the Y facilitates structural re-arrangements that can promote gene family expansion [25]. Additionally, the efficacy of natural selection is reduced on the non-recombining Y, and Y chromosomes across diverse taxa accumulate functionless and deleterious repetitive DNA [16]. Amplified Y genes thus may either provide no benefit for their carriers, or could in fact be slightly deleterious, yet natural selection is unable to remove them [26]. Heterochromatin formation on the Y may further dampen any functional consequences of gene family expansion, and multi-copy Y genes may simply be more tolerated on the silenced Y. Finally, some multi-copy Y genes may actually contribute to male fitness and fertility [12–14,27]. Gene family expansion on the Y chromosome may help to compensate for reduced gene dose on the heterochromatic and transcriptionally repressed Y chromosome [16,17]. Y chromosomes are transmitted from father to son, and are thus an ideal genomic location for genes that specifically enhance male fitness [28]. Y chromosomes of several species, including mammals and Drosophila, have been shown to contain multi-copy gene families that are expressed in testis and contribute to male fertility [12–14]. Our analysis shows that multi-copy Y genes are common across flies, and it will be of great interest to identify the diverse evolutionary processes driving their amplification.

Co-amplification of X/Y genes, on the other hand, is more difficult to explain under scenarios that do not involve genetic conflict. Several factors that may explain accumulation of genes on the Y do not apply to the X: The repeat content of X chromosomes is comparable to that of autosomes [22]; natural selection efficiently purges deleterious mutations from the recombining X; and transcription of the X chromosome in Drosophila males is increased in somatic tissues, rather than reduced [29]. In addition, co-amplified X and Y genes are enriched for meiosis functions (see also [24]), and the X-linked copies of co-amplified genes are highly expressed in testis [24]. Functions in chromatin formation and chromosome segregation might be expected for selfish genes that are trying to interfere with proper condensation of the heterochromatic Y chromosome, or with fair segregation of homologous chromosomes. Testis expression of co-amplified X-linked genes is unusual, as testis-expressed genes are underrepresented on the X chromosome of Drosophila ([30], but also see [31]), but can be understood under intragenomic conflict models [32–36]. Most importantly, production of double-stranded RNA and triggering of the RNAi pathway is inconsistent with gene amplification boosting gene product, but instead has the opposite effect and results in transcriptional down-regulation of co-amplified X/Y genes.

Could other evolutionary forces or properties of sex chromosomes account for co-amplification of sex-linked genes? In several species, X chromosomes are down-regulated during spermatogenesis. While there has been considerable debate about the exact mechanisms of male germline X inactivation in Drosophila, testis genes appear transcriptionally repressed during spermatogenesis on old X chromosomes in Drosophila [37–39]. Co-amplification of testis genes could compensate for reduced expression of inactivated sex-linked genes and may thus be an adaptation to counter silencing of sex-linked genes in spermatogenesis. However, this model would not explain why co-amplified genes are frequent targets by endo-siRNA [24], which instead indicates a conflict between the X- and Y-linked copies, and not coordinated selection for their up-regulation. Also, many copies of co-amplified genes are truncated (as we observe for *S-Lap* or *GAPsec*, but also for others; see S6 Table), suggesting that the duplicated copies do not have the same function as their parent copies. Gene amplification to counter male germline X inactivation would also predict that (co)-amplified genes are more abundant on older sex chromosomes, where inactivation of the X in spermatogenesis is complete. In contrast, we find that co-amplified genes are more common in species with young neo-sex chromosomes (Fig 1). In particular, the young neo-X chromosome of *D*. *miranda* shows no signs of reduced expression in testis [40], yet we detect the largest numbers of co-amplified genes in this species (Fig 1).

Co-amplification of X and Y-linked genes could also allow meiotic pairing between diverging sex chromosomes. In particular, the ribosomal RNA gene cluster is present on both the X and the Y in *D*. *melanogaster* and functions as an X-Y pairing site during male meiosis [41]. This model, however, would not explain meiosis-specific function and testis-expression of co-amplified genes, or their targeting by endo-siRNA. Also, acquiring homologous pairing sites should also be more important on more divergent, heteromorphic sex chromosomes, counter to our finding of co-amplified genes being more common on young neo-sex chromosomes.

### A model for co-amplification of sex-linked genes and meiotic drive

**Fig 6:**
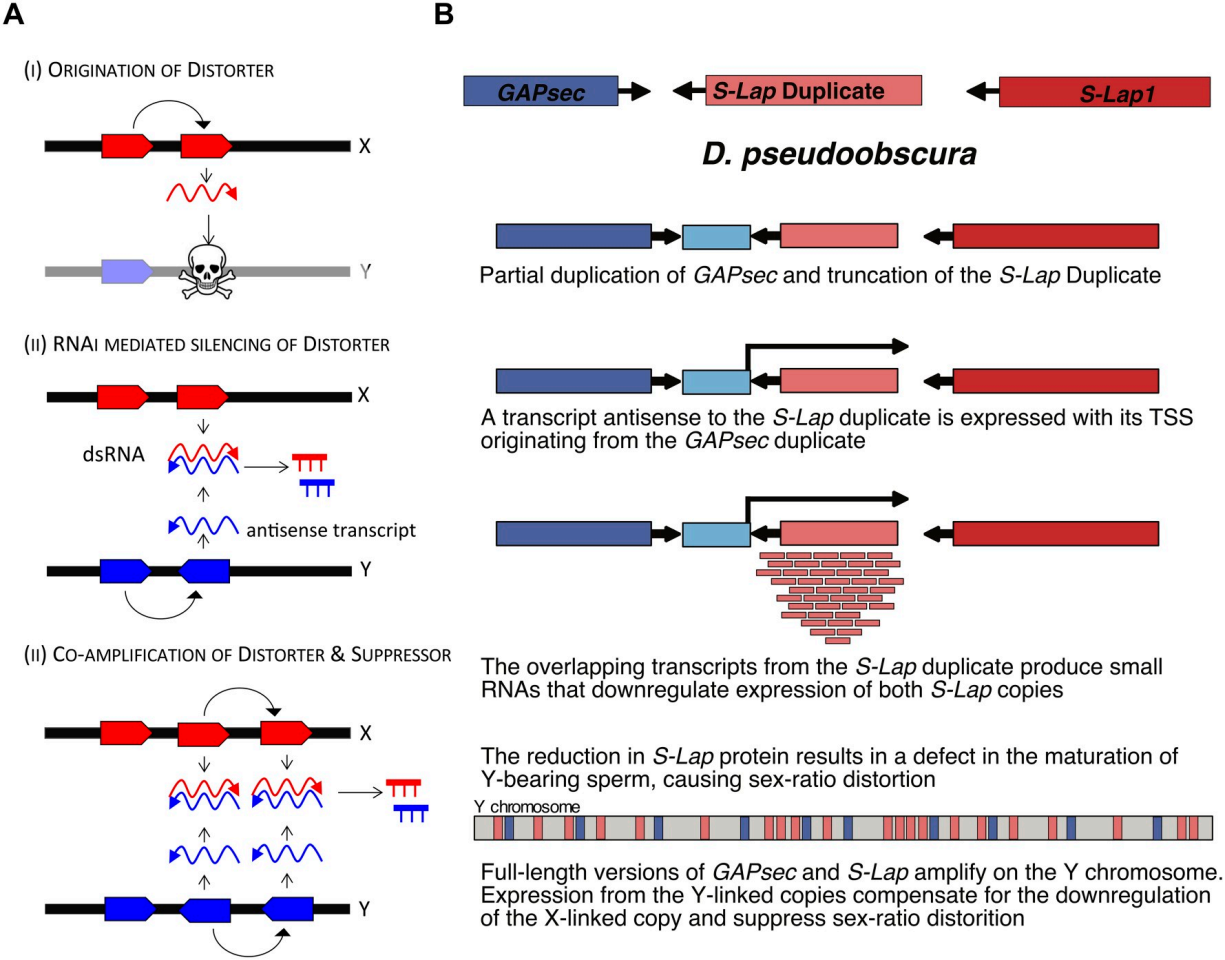
Meiotic conflict could fuel co-amplification of X and Y genes. **A**. A hypothetical model that may account for co-amplification of X- and Y genes and small RNA production invoking recurrent sex chromosome drive. An X-linked gene duplicate can evolve a novel function that eliminates Y bearing sperm. Amplification of the homologous Y gene, and production of antisense transcript may trigger the RNAi response, and silence the distorter. Repeated cycles of amplification of dosage-sensitive distorters and suppressors can result in the co-amplification of X/Y genes that are targeted by short RNAs. **B**. A hypothetical evolutionary model of the cryptic *S-Lap1* drive system. *S-Lap1* was duplicated in an ancestor of *D*. *pseudoobscura*, and a partial duplication of *GAPsec* created a TSS for anti-sense transcription of *S-Lap1* duplicate. Production of small RNA’s may deplete *S-Lap1* transcripts, which may result in elimination of Y-bearing sperm, and could be compensated by amplification of *S-Lap1* on the Y chromosome.

Co-amplification of genes on young sex chromosomes with meiosis-related functions, expression in testis, and targeting by endo-siRNAs can all be understood under a model of RNAi mediated cryptic sex chromosome drive. How would co-amplification of meiosis-related genes on the X and Y cause meiotic drive and its suppression? If amplified Y genes are involved in a battle with the X over fair transmission, changes in gene copy number may tip the balance over inclusion into functional sperm, and could result in repeated co-amplification of distorters and suppressors on the sex chromosomes (Fig 6A). In particular, an X-linked gene involved in chromosome segregation may evolve a duplicate that acquires the ability to incapacitate Y-bearing sperm (Fig 6A). Invasion of this sex-ratio distorter skews the population sex ratio and creates a selective advantage to evolve a Y-linked suppressor that is resistant to the distorter. Suppression may be achieved at the molecular level by increased copy number of the wildtype function or by inactivation of X-linked drivers using RNAi [33,34,42]. If both driver and suppressor are dosage sensitive, they would undergo iterated cycles of expansion, resulting in rapid co-amplification of both driver and suppressor on the X and Y chromosome [43]. Such a model is consistent with what we observe for the *S-Lap* and *GAPsec* genes in *D*. *pseudoobscura* (Fig 6B). *S-Lap1* is the most abundant sperm protein in *D*. *melanogaster* [18] but its function is poorly characterized. If this protein is crucial for generating Y-bearing sperm, depletion of *S-Lap1* during spermatogenesis would result in drive. *S-Lap1* was duplicated in an ancestor of *D*. *pseudoobscura*, and a partial duplication of *GAPsec* (and truncation of *S-Lap1*-duplicate) created a TSS for anti-sense transcription of *S-Lap1* duplicate. Anti-sense production of *S-Lap1-*duplicate transcript may trigger siRNA production and silencing of *S-Lap1*, which could result in elimination of Y-bearing sperm. Acquisition of multiple copies of S*-Lap1* on the Y chromosome could restore *S-Lap1* function, and create a cryptic drive system in *D*. *pseudoobscura* (Fig 6B). It is of course also possible that the Y-linked copies of *S-Lap1* interfere with the production of X-linked sperm (i.e. that the Y chromosome is the driver), and *S-Lap1*-duplicate on the X silences Y-copies through production of antisense RNA and RNAi. A similar model of cryptic drive could also explain patterns of molecular evolution and gene expression at *S-Lap* and *GAPsec* in *D*. *miranda*, where read-through transcription generates anti-sense transcripts that trigger RNAi. Detailed molecular testing will be necessary to characterize the wildtype function of *S-Lap1*, and the cellular basis of the putative drive phenotype and its suppression. Below, we discuss how several aspects of the co-amplified genes that we have identified would make sense under a model of sex chromosome drive.

### Co-amplified X/Y genes on neo-sex chromosomes

Most of the species where we identify co-amplified X/Y genes harbor neo-sex chromosomes. Under a drive model, this would make sense if sex ratio distorters have repeatedly evolved to exploit genomic vulnerabilities associated with the formation of new sex chromosomes. Different features of young vs. old sex chromosomes create different susceptibilities to sex chromosome drive. Old Y chromosomes are typically highly repetitive and heterochromatic, a feature that may easily be exploited by a driver on the X. Also, old sex chromosomes show much higher levels of sequence divergence, which makes identification and targeting of the homolog by a driver easier. Yet, young Y chromosomes typically contain many more genes that can evolve to cheat meiosis, thereby increasing the chances of a Y-linked driver. Finally, young X chromosomes may not yet be transcriptionally inactive during spermatogenesis and thus express more drivers. In many species, including Drosophila, expression from the X chromosome is reduced during spermatogenesis [37]. Low gene number and high repeat content makes Y chromosomes especially vulnerable to meiotic drive, and silencing of the X during spermatogenesis may have evolved as a genome defense against driving X’s [32]. Suppression of transcription during spermatogenesis may not have fully evolved on young X chromosomes, allowing the expression of more X-linked drivers. This may account for the prevalence of co-amplified X/Y genes in species with recently formed neo-sex chromosomes.

### Co-amplification and small RNAs

The RNAi pathway could be utilized in different ways to either create a meiotic driver, or to suppress it. For example, a gene on the X (or it’s duplicate) may gain a novel function that disrupts segregation of the Y chromosome. The homologous Y gene (or duplicates of it) may then silence the driving X by producing anti-sense transcripts that generate dsRNAs and launch the RNAi response, to silence the X-linked driver. This scenario resembles the Winters sex ratio system, even though the suppressors of X-linked drive are autosomal and not Y-linked [42]. The RNAi machinery can also be hijacked to create a driving X. In particular, if an X-linked gene is required for producing Y-bearing sperm, an X chromosome that silences this gene could evolve a drive phenotype. It could do so by antisense RNA production of this X-linked gene (or duplicates of it), thereby triggering the RNAi response to inactivate the gene. The organism could restore the wildtype function of this gene by increasing its dose through its amplification on the Y, and even non-functional copies may act as a decoy to soak up endo-siRNA that are targeting this locus. This pathway may underlie the putative *GAPsec* /*S-Lap1* drive system in *D*. *pseudoobscura*, where the X-linked duplicates of *S-Lap1* produce the vast majority of antisense transcripts (roughly 95%, see S8 Table), while the Y-linked *S-Lap1* copies predominantly generate sense RNA (>99.9%). Most of the small RNAs are produced from *S-Lap1* duplicate (the putative driver) and the Y-linked copies of *S-Lap1* (about 96% in total), consistent with the idea that amplification of this spermatogenesis gene allows restoration of wildtype function, possibly by acting as a decoy to dilute RNAi induced silencing triggered by antisense transcripts of *S-Lap1* duplicate. Under either scenario, if both the driver and suppressor are dosage-sensitive, this can lead to the repeated invasion of driving and suppressing chromosomes through co-amplification of genes on the X and Y chromosome.

In order to trigger the RNAi response, the production of dsRNA is required. This can be achieved in multiple ways. In the *D*. *simulans* Winters system, the two suppressor genes both encode related long inverted repeats that can form hairpin RNAs (hpRNAs), which are then processed by the RNAi machinery to generate siRNAs that repress the paralogous distorters [42]. Alternatively, the production of dsRNA can occur through anti-sense transcription of the target genes, and this mechanism is creating siRNAs in the putative drive involving *GAPsec* and *S-Lap1*, both in *D*. *pseudoobscura* and *D*. *miranda*.

### RNAi, young sex chromosomes and drive

Our data further support growing evidence that the production of antisense transcripts, hairpin RNAs and small RNAs may underlie some silenced meiotic drive systems [24,42]. RNA interference (RNAi) related pathways provide defense against viruses and transposable elements, and have been implicated in the suppression of meiotic drive elements [42]. Intriguingly, genes in these pathways often evolve rapidly, and show frequent gene duplication and loss over long evolutionary time periods. *Argonaute 2* (*Ago2*), for example, is one of the key RNAi genes in insects, and has repeatedly formed new testis-specific duplicates in the recent history of the *Drosophila obscura* group [44]. Analysis of additional RNAi-pathway genes confirms that they undergo frequent independent duplications and that their history has been particularly labile within the *Drosophila obscura* group [45]. Our finding suggests that the presence of young sex chromosomes in this species group makes them especially vulnerable to the invasion of meiotic drive elements, and may thus drive the rapid evolution of RNAi genes in this clade. It will be of interest to study the dynamics of RNAi genes in other species groups that have gained novel sex chromosomes, to see if diversification of RNAi genes is correlated with the emergence of new sex chromosomes.

It is important to note that RNAi is only one means by which co-amplified genes could compete with each other to create and suppress sex chromosome drive. In particular, drive and silencing for the well-studied, co-amplified *Slx*/*Sly* genes in mice involves competition of SLX and SLY at the protein level for entry to the nucleus and for nuclear binding sites [7,8][46]. Also, not all drive systems lead to gene amplification. The Winters sex-ratio driver in *D*. *simulans* encodes a duplicate X-linked distorter (*Dox*/*Nmy*) that is silenced by their paralogous autosomal suppressors *Nmy* and *Tmy* through RNAi [33,34,42], and the Paris sex-ratio drive in *D*. *simulans* drive is caused by deficient alleles of a fast-evolving X-linked heterochromatin protein, showing that the rapid evolution of genes involved in heterochromatin structure can fuel intragenomic conflict [47]. Careful molecular dissection of several drive systems is necessary to establish general characteristics of segregation distorters.

### X/Y copy number imbalance

Most co-amplified genes have considerably fewer copies on the X than the Y chromosome (S6 Table). Our approach is biased towards finding genes that are more highly amplified on the Y relative to the X, since we require genes to have increased M/F coverage ratios to be classified as Y-amplified in the first place. However, analyses of high-quality genome assemblies of *D*. *pseudoobscura* and *D*. *miranda* confirm that co-amplified genes indeed have many fewer copies on the X than on the Y, in both species (average copy number is 3 on the X, versus 44 on the Y). Thus, this difference in copy number of co-amplified genes on the X and the Y is not simply an artifact and surprising under a simple model of dose-dependent co-amplification of meiotic drivers and their suppressors. The reasons for this difference are not clear, but could include the following: Expression on the heterochromatic Y is generally dampened, and disproportionately more copies of a gene are needed to balance amplified X genes. This is supported by gene expression data from *S-Lap1*, where one parental copy on XR produces almost as many transcripts as we observe from the dozens of Y-linked copies combined (see S8 Table). Additionally, high copy-number gene arrays may be more difficult to maintain on the recombining X chromosome [48]. Also, RNAi induced drive models may not be stoichiometric. If X-linked drive operates by inactivation of a gene essential for Y chromosomes through antisense RNA production, a much larger number of sense Y-linked transcripts may be required to dilute silencing antisense transcripts. Interestingly, co-amplified genes on the mouse sex chromosomes are also much more abundant on the Y relative to the X; *Sly*/*Slx* have 126 copies on the Y and 39 on the X, *Ssty*/*Sstx* have 306 copies on the Y and 11 on the X, and *Srsy*/*Srsx* have 197 copies on the Y and 14 on the X [49]. Thus, higher copy number on the Y appears to be a general feature of co-amplified X/Y genes.

### *S-Lap* and *GAPsec* as putative drivers in *pseudoobscura* group flies

It is intriguing that *S-Lap* and *GAPsec* have repeatedly and independently co-amplified on the X and Y of multiple species in the *D*. *obscura* group, where Muller element D became sex-linked. In both *D*. *pseudoobscura* and *D*. *miranda*, two species for which we have stranded testis RNA-seq data and small RNA profiles, both genes produce endo-siRNA, indicating their involvement in a genomic conflict (see above). Understanding the molecular basis of this putative drive system will require detailed experimental work to characterize the wild-type function of these genes during spermatogenesis in the *D*. *obscura* group, and careful manipulation of the co-amplified X and Y copies. The function of *S-Lap* is poorly studied, but a recent paper suggests that all *S-Lap* genes in *D*. *melanogaster* are structural components of the mitochondrial paracrystalline material in sperm tails [50]. Sperm tail morphology varies dramatically between *D*. *melanogaster* and *D*. *pseudoobscura*, and unlike *D*. *melanogaster*, *D*. *pseudoobscura* has two types of sperm: long fertile eusperm (which are roughly 400μm long), and short infertile parasperm (roughly 100μm long; [51] [52]). While sperm heteromorphism is an intriguing phenomena, and may be exploited for drive, it complicates comparisons between sperm morphology and function between *D*. *melanogaster* and *D*. *pseudoobscura*. Intriguingly, *GAPsec* is a GTPase activating protein (*GAPse* is a Rab-GTPase), similar to the well-characterized *Sd* gene in *D*. *melanogaster*, which is a truncated duplicate of the *RanGAP* gene (which is a Ran-GTPase, [19]). Thus, either (or both) gene(s) may be considered a good candidate to be involved in meiotic drive, and it will be of great interest to study the wildtype function of these genes during spermatogenesis in *D*. *pseudoobscura* and its relatives. We focused our analysis in *D*. *pseudoobscura* on *S-Lap1*, since its duplicate copy on the X is a readily detectable and annotated gene that is transcribed. The duplicate of *GAPsec*, on the other hand, is highly degraded in *D*. *pseudoobscura*, not annotated as a gene, and it shows low levels of transcription. However, the fact that *GAPsec* is a GTPase activator that is independently co-amplifying with *S-Lap* on both the X and the Y in different species is captivating, and we readily detect antisense transcripts and small RNAs from both genes in *D*. *miranda*. This could mean that antisense transcription of either gene or possibly both is required for drive, and it could also imply that the cryptic drive system in *D*. *pseudoobscura* is now defunct. Higher copy number of both *S-Lap* and *GAPsec* on the Y, and more divergence among Y copies in *D*. *pseudoobscura* compared to *D*. *miranda* is consistent with this cryptic drive being older in *D*. *pseudoobscura*. Enough time may have passed in *D*. *pseudoobscura* for the drive to be fully silenced, after which point there is no selection to retain the driver, and it may start accumulating deactivating mutations.

To conclude, our comparative analysis suggests that co-amplification of genes on X and Y chromosomes may be relatively common in Drosophila, especially on young sex chromosomes, and we have shown that the same genes have been independently co-amplified in multiple species from the *obscura* group. We considered several evolutionary scenarios that would explain such amplifications, including compensation for male germline X inactivation, the formation of gene arrays to aid in meiotic chromosome pairing, and sex chromosome drive. We believe that sex chromosome drive is the most likely explanation for this pattern, for reasons discussed above, however, proof of this hypothesis will require careful experimental validation. The fact that these genes exist in multiple copies, are highly similar on the X and Y, and were all found in non-model Drosophila species that lack transgenic resources will make experimental validation of cryptic drive a very difficult task. That said, future characterization of the putative drive systems identified here would provide a full picture of how distorting elements manipulate and cheat meiosis, what molecular pathways or developmental processes are particularly vulnerable, and how the genome has launched evolutionary responses to counter distortion.

## Methods

### Genome sequencing & assembly

Strains were acquired from the Drosophila Species Stock Center (UC San Diego) or the EHIME stock center (Ehime University, Japan) as indicated in S1 Table. For each strain, DNA was extracted from a single male and a single female, using the Qiagen Gentra Puregene cell kit. The Illumina TruSeq Nano DNA library preparation kit was used to prepare 100 bp paired-end sequencing libraries for all species except *D*. *robusta*, *D*. *melanica*, and *D*. *willistoni*. For these species, the Illumina Nextera DNA library preparation kit was used to prepare 150 bp paired-end sequencing libraries. The genome assemblies produced for this study are noted in S1 Table. Assemblies were produced from the female data: reads were error-corrected using BFC [53] and assembled using IDBA-UD [54] with default parameters.

### Identification of X-A fusions

X chromosome/autosome fusions were identified in two steps [55]. For each species, genomic scaffolds were assigned to Muller elements based on their gene content, inferred from the results of a translated BLAST search of *D*. *melanogaster* peptides to the assembly of interest. Scaffolds smaller than 5kb were excluded. Next, the male and female Illumina data were separately mapped to the female assembly using Bowtie2 [56] and excluding alignments with mapping quality less than 20. The coverage ratio (M/F) was calculated for each scaffold that was assigned to a Muller element. The distribution of coverage ratios for each Muller element (S1 Fig) was then examined to determine if any of the ancestral autosomes had become X-linked. The raw (un-normalized) ratios are reported in S1 Fig. Most libraries were sequenced with similar number of reads for both males and females but for others, there was more data for males. Regardless of the value itself, the M/F values for an X-linked chromosome should be approximately half of the Y-linked values.

### Identification of co-amplified genes on the X- and Y-chromosome

To characterize co-amplified genes on the sex chromosomes, we first identify genes amplified on the Y. For each species, male and female Illumina reads were separately aligned to a filtered version of the *D*. *melanogaster* peptide set, where only the longest isoform of each gene was retained. To generate these alignments, the DIAMOND software package [57] was used to perform a translated search of each Illumina read to the peptide set. Read coverage for each peptide sequence was calculated in 30 amino acid non-overlapping windows and normalized by dividing by the total number of mapped reads. The M/F coverage ratio was computed by dividing the median male coverage by the median female coverage, for each peptide. We required that potentially Y-amplified genes have a normalized M/F coverage ratio of at least 2.5 and only retained genes whose parent copy was X-linked in the species of interest. We searched for X-linked duplicates in the female genome assemblies by first using Exonerate [58] to extract the coding sequence of the best hit between the *D*. *melanogaster* peptide and the female assembly. We then used BLASTN [59] to obtain a stringent (E-value threshold = 1e-20) list of all non-overlapping hits between each exon of the coding sequence and the genome assembly. We considered a gene to be duplicated in females if at least 25% of the parent coding sequence aligned to more than one location in the genome assembly.

### *S-Lap1* and *GAPsec* gene trees and Y chromosome gene copies

The Muller-D copies of *S-Lap1* and *GAPsec* were identified in the 12 Drosophila genomes [60] by synteny with *D*. *melanogaster* and their coding sequences were downloaded from FlyBase [61]. The PRANK software package [62] was used to generate codon-aware alignments of coding sequences for each gene. The resulting alignment was trimmed using trimAl [63] and RaxML [64] was used to infer a maximum likelihood phylogeny (100 bootstrap replicates). *D*. *pseudoobscura* Y-linked contigs were identified using read coverage information from male versus female genomic sequencing data. Exonerate [58] was used to determine the location of the amplified copies of *S-Lap1* and *GAPsec* on these scaffolds with the *D*. *pseudoobscura S-Lap1* (*FBpp0285960*) and *GAPsec* (*FBpp0308917*) peptide sequences as queries. The *D*. *pseudoobscura* Y copies of each gene were aligned using MAFFT [65], trimmed with trimAl [63], and a Y consensus sequence for each gene was generated using PILER [66].

### RNA libraries and mapping

We dissected testes from 3–8 day old virgin males of *D*. *pseudoobscura* (strain MV25) reared at 18°C on Bloomington food. We used Trizol (Invitrogen) and GlycoBlue (Invitrogen) to extract and isolate total RNA. *D*. *pseudoobscura* CAGE-seq data were obtained from the ModEncode project [67]. We resolved 20 μg of total RNA on a 15% TBE-Urea gel (Invitrogen) and size selected 19–29 nt long RNA, and used Illumina’s TruSeq Small RNA Library Preparation Kit to prepare small RNA libraries, which were sequenced on an Illumina HiSeq 4000 at 50 nt read length (single-end). We used to Ribo-Zero to deplete ribosomal RNA from total RNA, and used Illumina’s TruSeq Stranded Total RNA Library Preparation Kit to prepare stranded testis RNA libraries, which were sequenced on an Illumina HiSeq 4000 at 100 nt read length (paired-end). Total RNA data were aligned to the *D*. *pseudoobscura* reference genome using HISAT2 [68], whereas Bowtie2 [56] (seed length: 18) was used to align small RNA and CAGE-seq data. In all cases, alignments with mapping quality less than 20 were discarded.

## Supporting information

S1 Fig Identification of newly formed sex chromosomes across Drosophila. Sex chromosomes are inferred using male and female coverage data. Plotted is the male / female genomic read coverage for scaffolds mapped to the *D*. *melanogaster* genome, to infer the location of Muller elements.
(PDF)

S2 Fig Bioinformatic identification of co-amplified X/Y genes. Multicopy Y genes are identified based on mapping of male and female genomic reads to D. melanogaster proteins using translated BLAST searches. Multi-copy X-linked homologs are identified for multi-copy Y genes, based on genome assemblies. Sex-linkage of contigs is inferred based on male and female read coverage of contigs, or based on published genome assemblies for a subset of species.
(PDF)

S3 Fig Validation of bioinformatics pipeline to infer multi-copy Y genes in *D*. *miranda*. Shown is the predicted coverage based on mapping of Illumina reads on the x-axis, versus the number of Y-linked copies of a gene found in the genome assembly (Spearman’s rho: 0.77; p = 0.0008). Note that our bioinformatics pipeline is conservative and underestimates the number of Y-linked copies found in the assembly, presumably due to many multi-copy genes being fragmented in the assembly.
(PDF)

S4 Fig Chromosomal location of multi-copy Y genes. All genes that showed evidence of multiple copies on the Y chromosome were assigned to chromosome arms based on their homologs in *D*. *melanogaster*. The Y-amplified genes from each species were then categorized based on their chromosome of origin. Species with a neo-X chromosome are denoted by the black horizontal bars.
(PDF)

S5 Fig GO functions of A. co-amplified X/Y genes and B. multi-copy Y genes. (PDF)

S6 Fig Alignment of X-linked and Y-linked copies of A. *S-Lap1* and B. *GAPsec*. (PDF)

S7 Fig Size distribution of short RNAs mapping to X and Y linked copies of A. *S-Lap1* and B. *GAPsec*. (PDF)

S8 Fig Mappability of RNA-seq and short RNA data to X-linked and Y-linked copies of *S-Lap1* and *GAPsec*. Panels A & E: Overview of alignment between the two X-linked copies of S-Lap/GAPsec and the consensus of the Y-linked copies. Panels B & F: Percent Identity between pairwise S-Lap/GAPsec alignments, calculated in 50 bp non-overlapping windows. Panel C & G: S-Lap/GAPsec Illumina sequence mappability. Grey shading shows locations where sequence reads of length > = 18 bp (small RNA) or > = 100 bp (RNA-seq) align uniquely when mapped to the full genome assembly. Panels D & H: Length distribution of Y-linked copies of S-Lap/GAPsec.
(PDF)

S9 Fig Mapping of short RNAs to testis transcripts in *D*. *pseudoobscura*. (PDF)

S1 Table Species used in this study. Shown are species and stock numbers, total assembly size, and sex chromosome karyotype (see S1 Fig).
(PDF)

S2 Table Number of amplified Y genes. Shown are the number of inferred amplified Y-linked genes found in each species, for a cut-off of male/female coverage ratio (M/F) > = 2.5.
(PDF)

S3 Table Amplified Y genes vs. M/F cutoffs. Showns are the numbers of amplified Y genes identified, for different cut-offs of male/female coverage ratio (M/F from 2.5 to 10).
(PDF)

S4 Table Multi-copy Y-linked genes across Drosophila species. Shown are the orthologous location of multi-copy Y genes in *D*. *melanogaster*, and their inferred molecular function and gene expression pattern in *D*. *melanogaster* (data from flybase.org).
(PDF)

S5 Table Inferred copy numbers for co-amplified X and Y genes. (PDF)

S6 Table Comparison of inferred copy numbers of co-amplified X and Y genes. Shown are inferred copy numbers of co-amplified X and Y genes, based on our analysis, and compared to high-quality genome assemblies from *D*. *miranda* and *D*. *pseudoobscura*.
(PDF)

S7 Table GO functions of multi-copy Y genes, and genes co-amplified on the X and Y. (PDF)

S8 Table Mapping of total RNA and short RNA for X-linked and Y-linked copies of *GAPsec* and *S-Lap1*. (PDF)

S9 Table Genes showing antisense transcription and targeting by short RNAs in *D*. *pseudoobscura* testis. Shown is the expression of the sense and antisense transcript, and expression of small RNA, and the location of paralogs in the *D*. *pseudoobscura* genome. Gene expression data are given in S5 Data.
(PDF)

S1 Data *GAPsec* sequence alignment (for phylogeny in Fig 2). (FASTA)

S2 Data *S-Lap* sequence alignment (for phylogeny in Fig 2). (FASTA)

S3 Data *GAPsec* sequence alignment (for phylogeny in Fig 3). (FASTA)

S4 Data *S-Lap* sequence alignment (for phylogeny in Fig 3). (FASTA)

S5 Data Total RNA and small RNA expression in *D*. *pseudoobscura* testis. (TAB)

S6 Data *GAPsec* sequence alignment (for phylogeny in Fig 5). (FASTA)

S7 Data *S-Lap* sequence alignment (for phylogeny in Fig 5). (FASTA)
