# Supplementary material for: Recurrent gene co-amplification on Drosophila X and Y chromosomes
Source: PLoS Genet. 2019 Jul 22;15(7):e1008251. doi: 10.1371/journal.pgen.1008251 (PMC6690552; doi:10.1371/journal.pgen.1008251)
Supplement: S1 Fig — Sex chromosomes are inferred using male and female coverage data. Plotted is the male / female genomic read coverage for scaffolds mapped to the D. melanogaster genome, to infer the location of Muller elements. (PDF) [file pgen.1008251.s001.pdf]

I. Infer Y-amplified genes based on Genomic read coverage

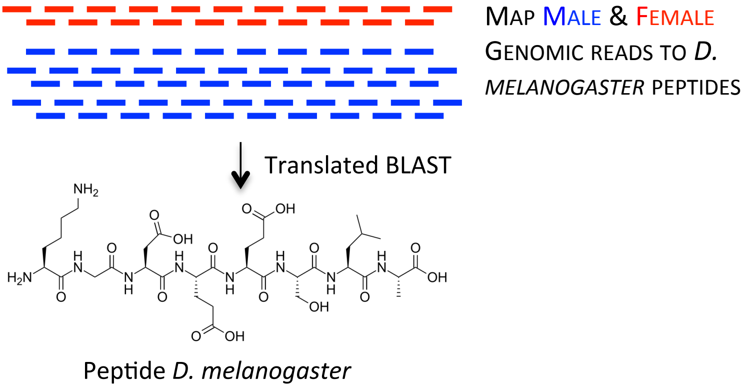

II. Identify X-linked contigs and Multi-copy X genes in Female Assembly

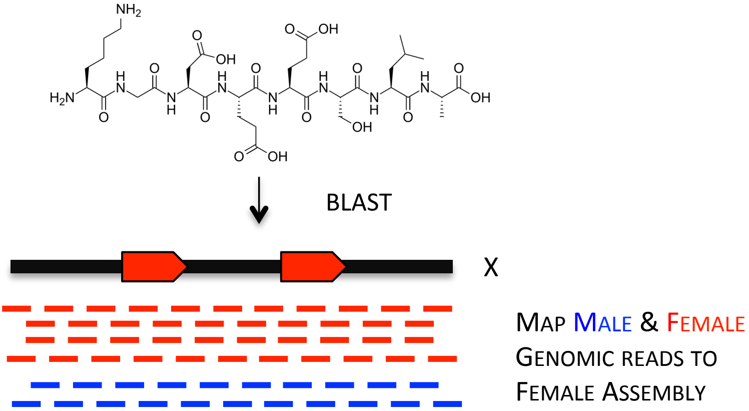

Figure S1
