# Supplementary figures and images for: Recurrent gene co-amplification on Drosophila X and Y chromosomes
Source: PLoS Genet. 2019 Jul 22;15(7):e1008251. doi: 10.1371/journal.pgen.1008251 (PMC6690552; doi:10.1371/journal.pgen.1008251)

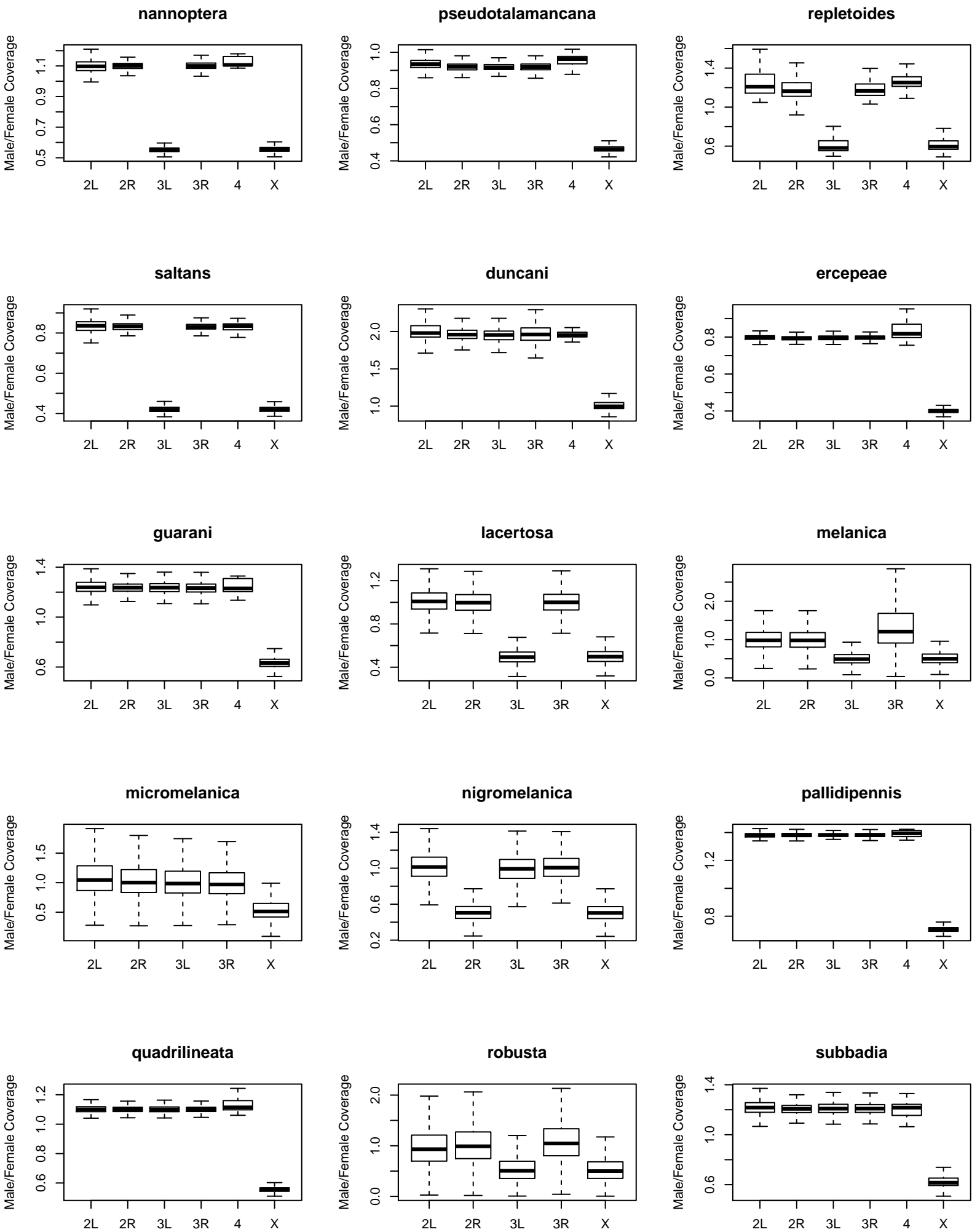

Figure S2.

Supplement: S2 Fig — Multicopy Y genes are identified based on mapping of male and female genomic reads to D. melanogaster proteins using translated BLAST searches. Multi-copy X-linked homologs are identified for multi-copy Y genes, based on genome assemblies. Sex-linkage of contigs is inferred based on male and female read coverage of contigs, or based on published genome assemblies for a subset of species. (PDF) [file pgen.1008251.s002.pdf]

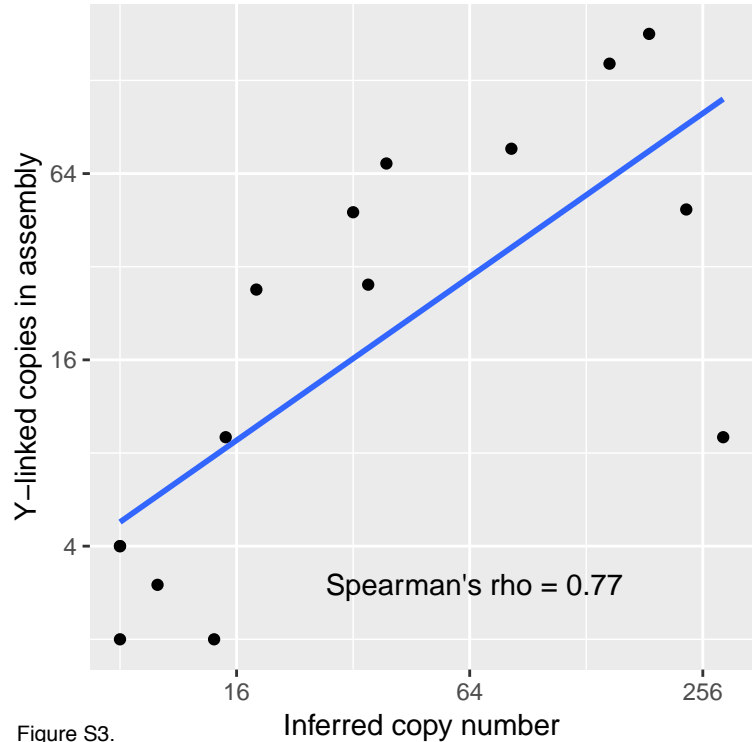

Figure S3.

Supplement: S3 Fig — Shown is the predicted coverage based on mapping of Illumina reads on the x-axis, versus the number of Y-linked copies of a gene found in the genome assembly (Spearman’s rho: 0.77; p = 0.0008). Note that our bioinformatics pipeline is conservative and underestimates the number of Y-linked copies found in the assembly, presumably due to many multi-copy genes being fragmented in the assembly. (PDF) [file pgen.1008251.s003.pdf]

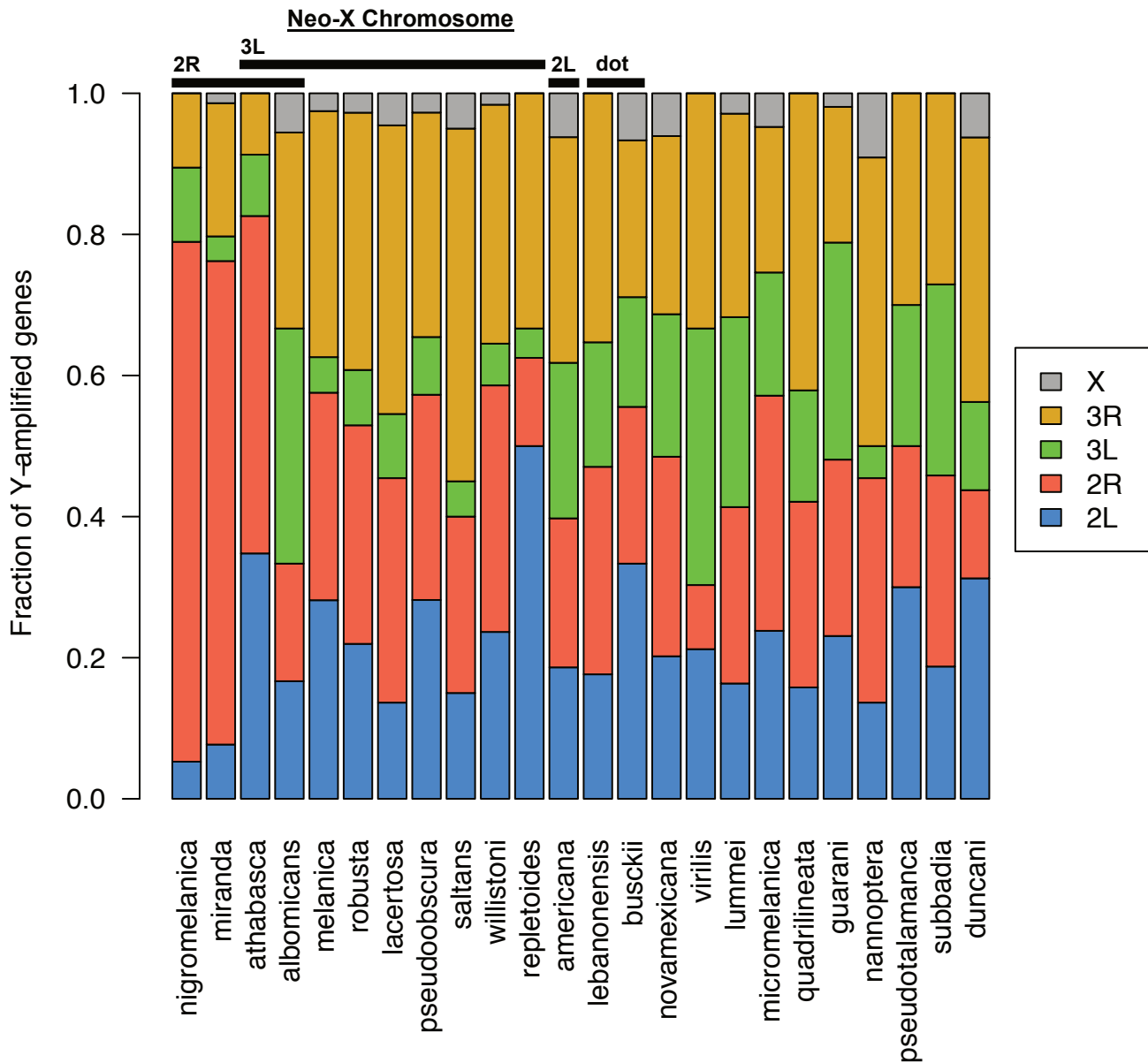

Figure S4.

Supplement: S4 Fig — All genes that showed evidence of multiple copies on the Y chromosome were assigned to chromosome arms based on their homologs in D. melanogaster. The Y-amplified genes from each species were then categorized based on their chromosome of origin. Species with a neo-X chromosome are denoted by the black horizontal bars. (PDF) [file pgen.1008251.s004.pdf]

$> 10^{-3}$

$10^{-3}$  to  $10^{-5}$

$10^{-5}$  to  $10^{-7}$

$10^{-7}$  to  $10^{-9}$

$< 10^{-9}$

A.

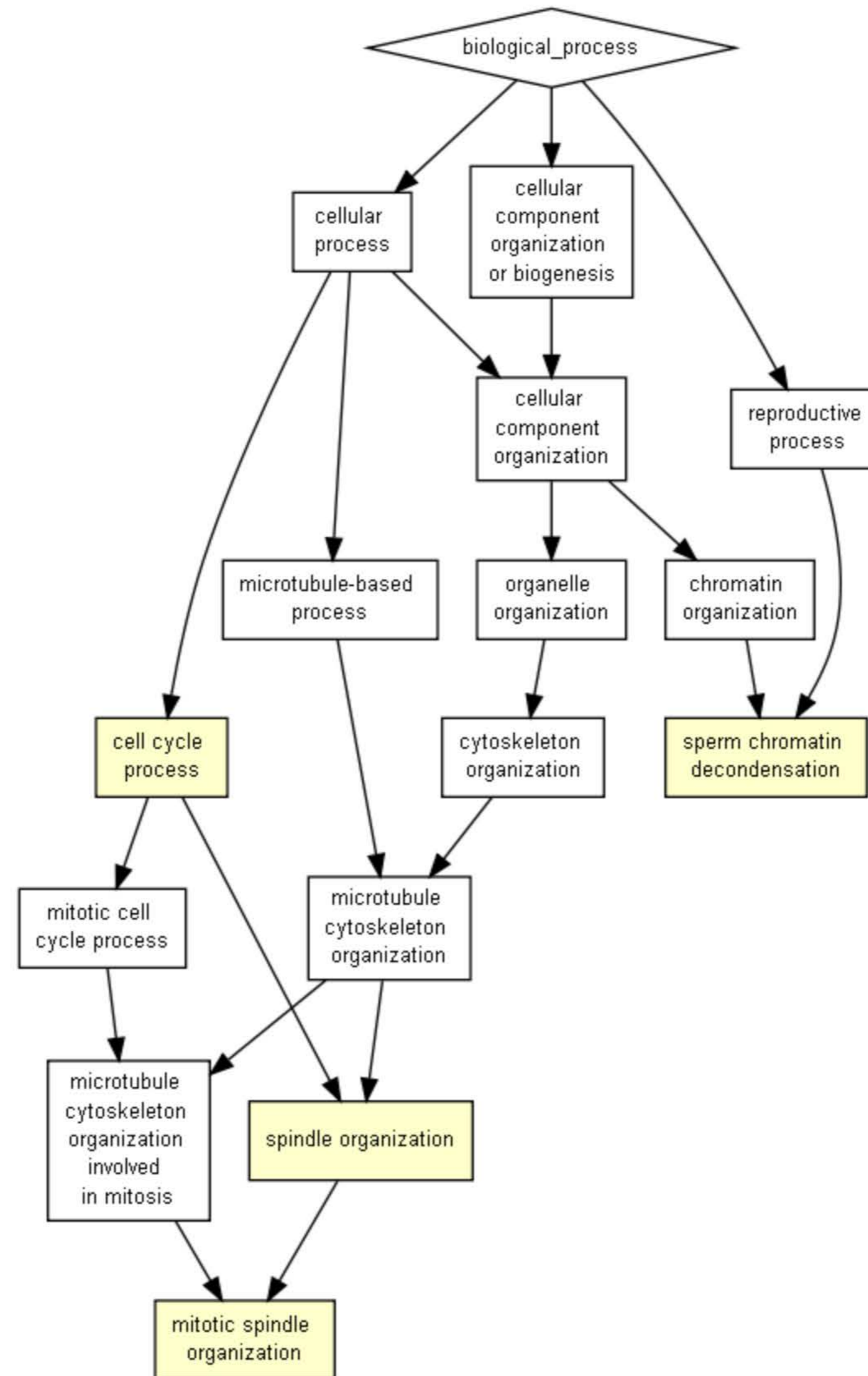

Figure S5

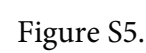

Supplement: S5 Fig — (PDF) [file pgen.1008251.s005.pdf]

**A**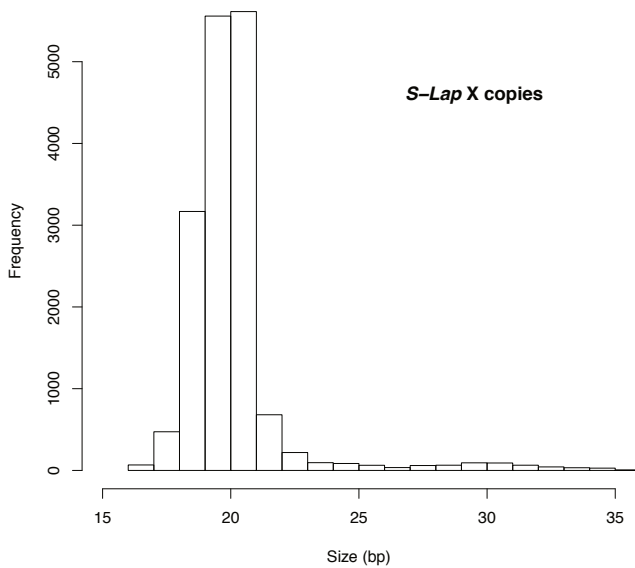**B**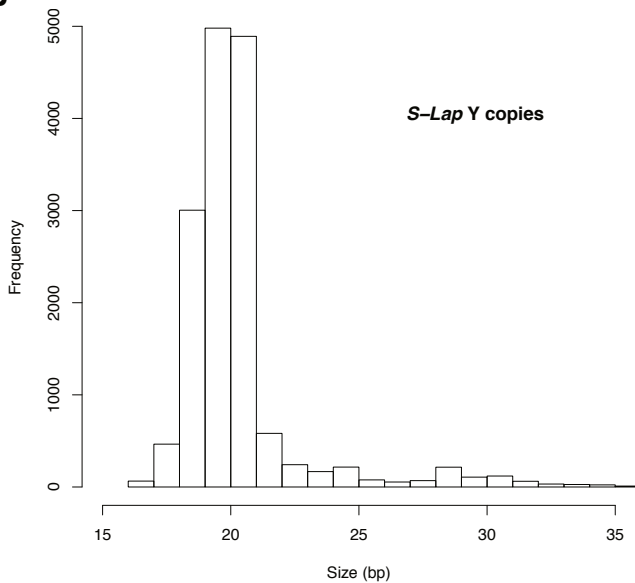**Figure S7**

**C**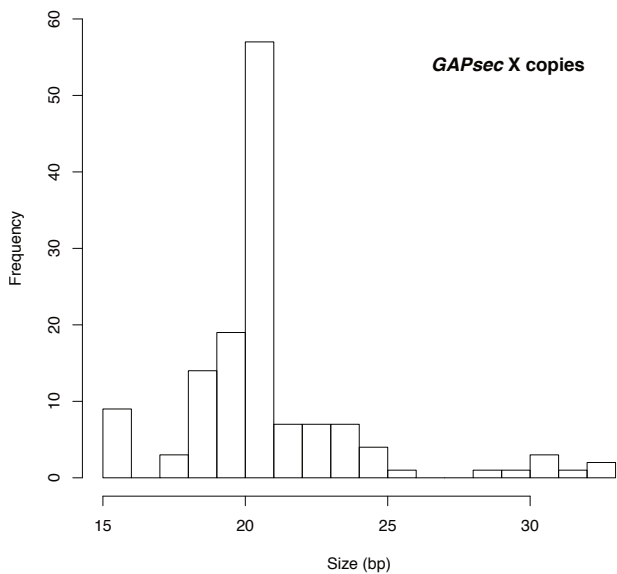**D**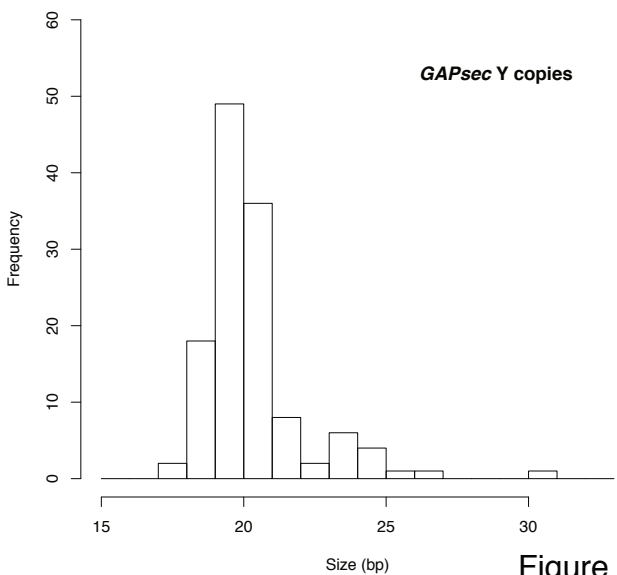**Figure S7**

Supplement: S7 Fig — (PDF) [file pgen.1008251.s007.pdf]

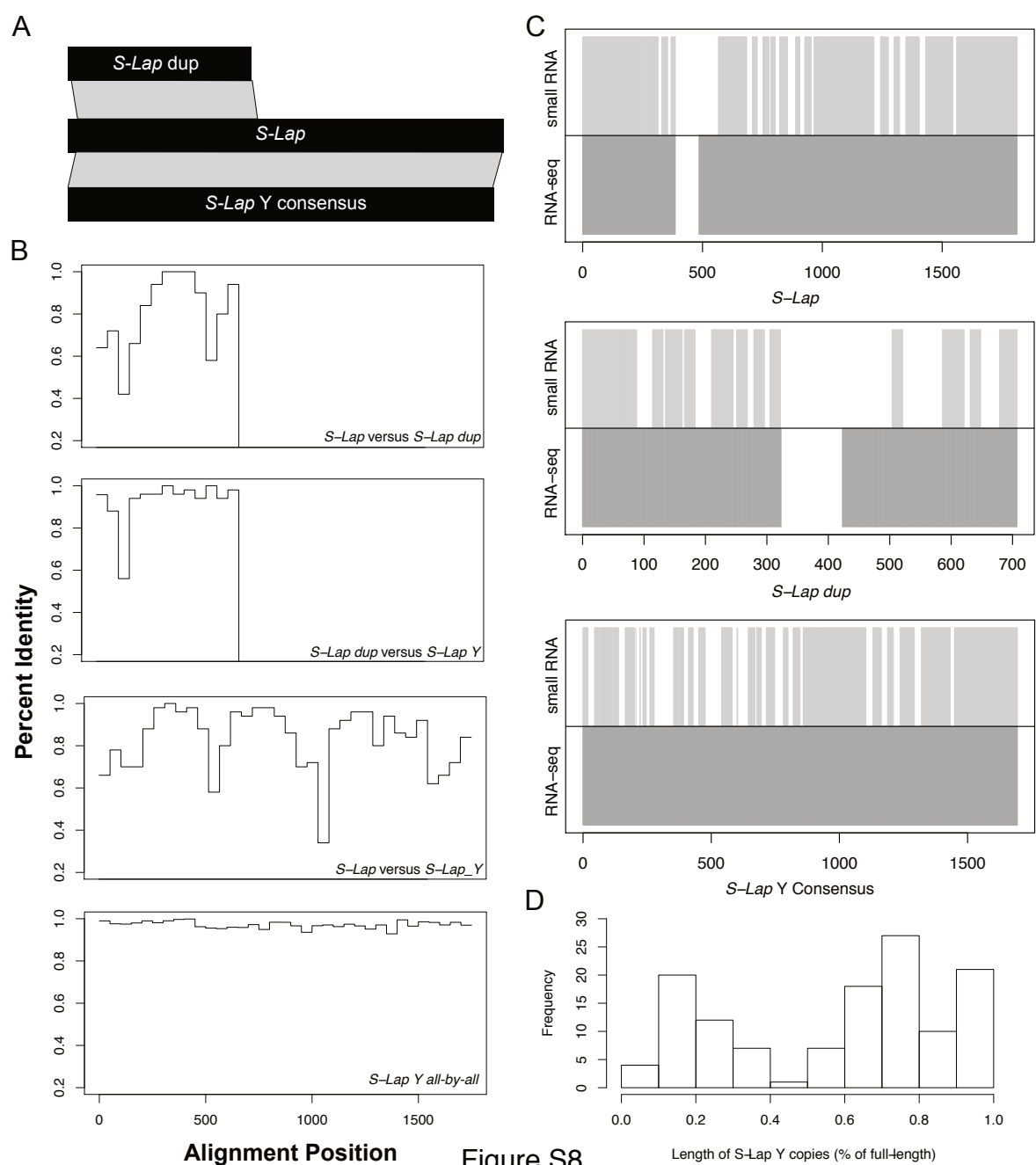

Figure S8

E

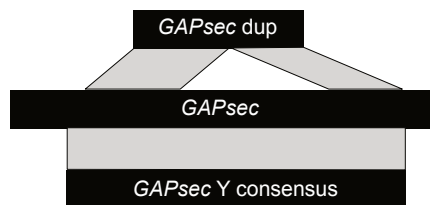

F

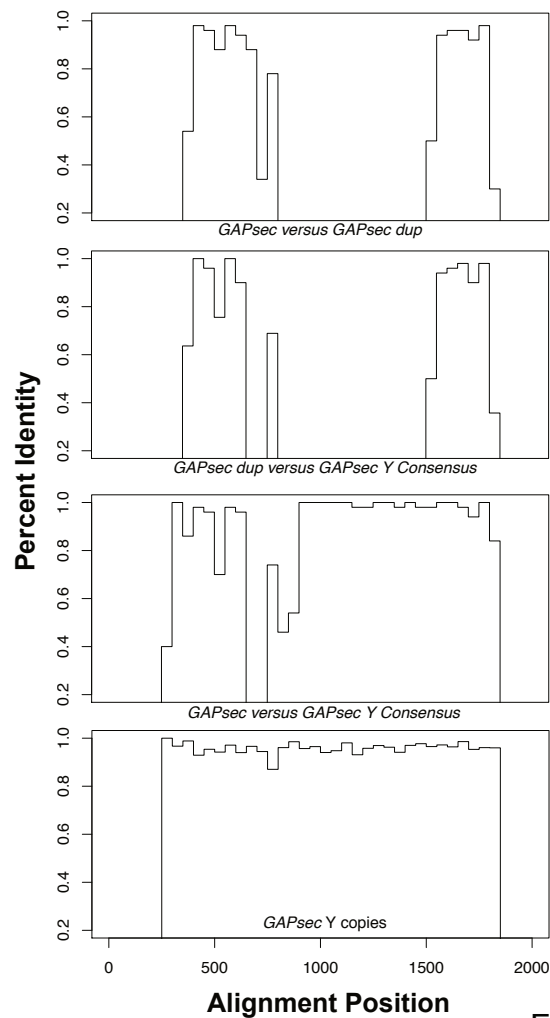

G

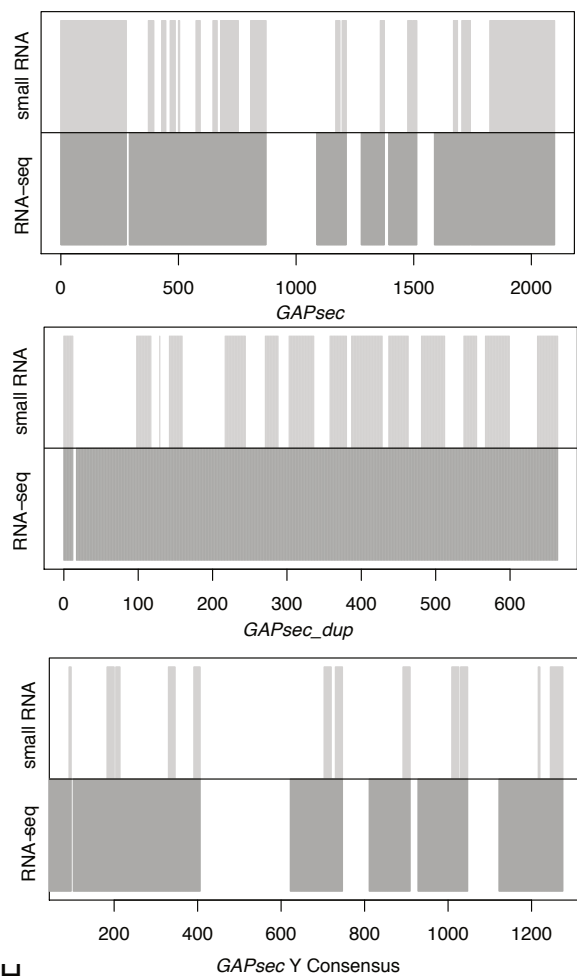

H

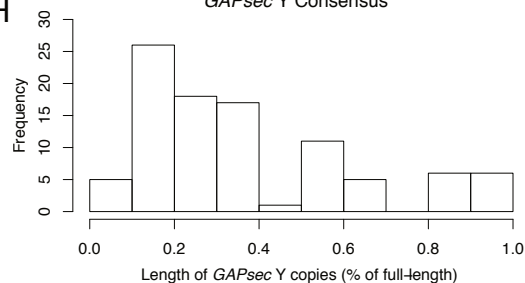

Figure S8

Supplement: S8 Fig — Panels A & E: Overview of alignment between the two X-linked copies of S-Lap/GAPsec and the consensus of the Y-linked copies. Panels B & F: Percent Identity between pairwise S-Lap/GAPsec alignments, calculated in 50 bp non-overlapping windows. Panel C & G: S-Lap/GAPsec Illumina sequence mappability. Grey shading shows locations where sequence reads of length > = 18 bp (small RNA) or > = 100 bp (RNA-seq) align uniquely when mapped to the full genome assembly. Panels D & H: Length distribution of Y-linked copies of S-Lap/GAPsec. (PDF) [file pgen.1008251.s008.pdf]

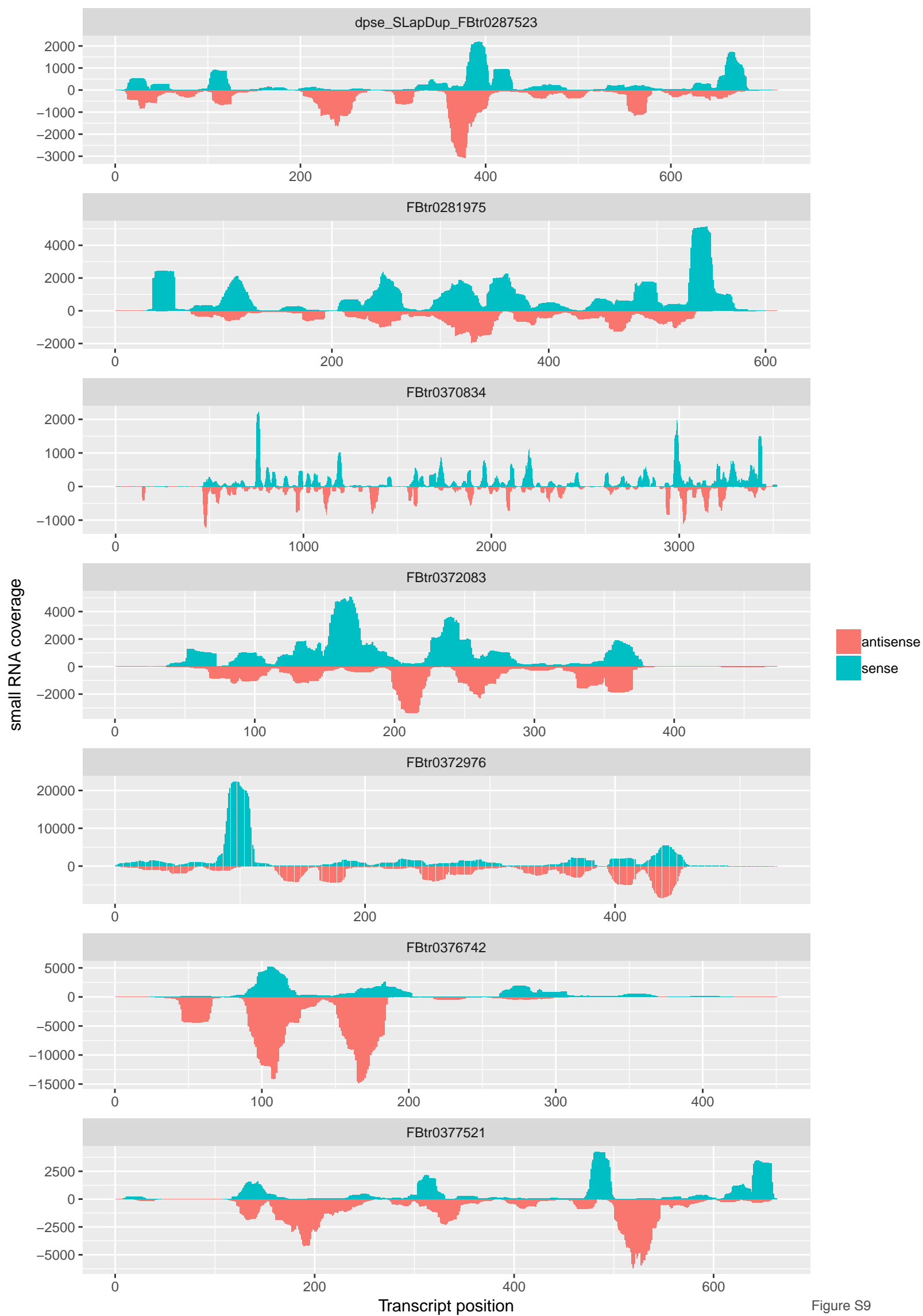

Supplement: S9 Fig — (PDF) [file pgen.1008251.s009.pdf]
