# Supplementary material for: Recurrent gene co-amplification on Drosophila X and Y chromosomes
Source: PLoS Genet. 2019 Jul 22;15(7):e1008251. doi: 10.1371/journal.pgen.1008251 (PMC6690552; doi:10.1371/journal.pgen.1008251)
Supplement: S1 Table — Shown are species and stock numbers, total assembly size, and sex chromosome karyotype (see S1 Fig). (PDF) [file pgen.1008251.s010.pdf]

Table S1. Species used in this study. Shown are species and stock numbers, total assembly size, and sex chromosome karyotype (see Figure S1).

Data generated for this study					
**Genus**	**Species**	**Stock Center**	**Stock Number**	**Assembly**	**Karyotype**
Drosophila	saltans	UCSD	14045-0911.01	190 Mb	A + D
Drosophila	nannoptera	UCSD	15090-1692.12	135 Mb	A + D
Drosophila	pseudotalamanca	UCSD	15040-1191.01	146 Mb	A
Drosophila	repletoides	UCSD	15250-2451.00	166 Mb	A + D
Drosophila	robusta	UCSD	15020-1111.10	184 Mb	A + D
Drosophila	micromelanica	UCSD	15030-1151.01	146 Mb	A
Drosophila	melanica	UCSD	15030-1141.03	150 Mb	A + D
Drosophila	nigromelanica	EHIME	E-22901	164 Mb	A + C
Drosophila	lacertosa	EHIME	E-14007	155 Mb	A + D
Scaptodrosophila	lebanonensis	UCSD	11010-0011.00	215 Mb	A + F
Drosophila	subbadia	UCSD	15172-2161.00	174 Mb	A
Drosophila	guarani	UCSD	15172-2151.00	170 Mb	A
Drosophila	quadrilineata	EHIME	E-14402	166 Mb	A
Drosophila	ercepeae	UCSD	14024-0432.00	156 Mb	A
Hirtodrosophila	duncani	UCSD	92000-0075.00	205 Mb	A
Drosophila	pallidipennis	UCSD	15210-2331.01	167 Mb	A
Drosophila	virilis	UCSD	15010-1051.46	166 Mb	A

Published Data		**Genome Source**	**Male Illumina Reads**	**Female Illumina Reads**	
Drosophila	pseudoobscura	FlyBase	PMID: 25879221	PMID: 25879221	A + D
Drosophila	athabasca	PMID: 28431021	PMID: 28431021	PMID: 28431021	A + D
Drosophila	miranda	PMID: 22822149	PMID: 25879221	PMID: 25879221	A + D + C
Drosophila	albomicans	PMID: 22439699	PMID: 25879221	PMID: 25879221	A + D + C
Drosophila	novomexicana	This Study	PMID: 28739599	This Study: UCSD 15010-1031.00	A
Drosophila	americana	This Study	PMID: 28739599	This Study: UCSD 15010-1041.00	Male: A + B
Drosophila	lummei	This Study	PMID: 28739600	This Study: UCSD 15010-1011.09	A
Drosophila	busckii	PMID: 26114585	PMID: 25879221	PMID: 25879221	A + F
Drosophila	willistoni	FlyBase	This Study: 14030-0811.24	This Study: 14030-0811.24	A + D
