# Supplementary material for: Recurrent gene co-amplification on Drosophila X and Y chromosomes
Source: PLoS Genet. 2019 Jul 22;15(7):e1008251. doi: 10.1371/journal.pgen.1008251 (PMC6690552; doi:10.1371/journal.pgen.1008251)
Supplement: S2 Table — Shown are the number of inferred amplified Y-linked genes found in each species, for a cut-off of male/female coverage ratio (M/F) > = 2.5. (PDF) [file pgen.1008251.s011.pdf]

Table S2. Number of amplified Y genes. Shown are the number of inferred amplified Y-linked genes found in each species, for a cut-off of male/female coverage ratio (M/F) >= 2.5

species	no. of amplified Y genes	Karyotype
D. duncani	16	A
D. ercepeae	8	A
D. guarani	53	A
D. lummei	109	A
D. micromelanica	63	A
D. novamexicana	105	A
D. pallidipennis	8	A
D. pseudotalamanca	10	A
D. quadrilineata	20	A
D. subbadia	48	A
D. virilis	35	A
D. americana	323	A + B
D. nigromelanica	38	A + C
D. athabasca	24	A + D
D. lacertosa	23	A + D
D. melanica	249	A + D
D. nannoptera	22	A + D
D. pseudoobscura	111	A + D
D. repletoides	25	A + D
D. robusta	265	A + D
D. saltans	20	A + D
D. willistoni	187	A + D
D. albomicans	30	A + D + C
D. miranda	143	A + D + C
D. busckii	48	A + F
D. lebanonensis	17	A + F
