# Supplementary material for: Recurrent gene co-amplification on Drosophila X and Y chromosomes
Source: PLoS Genet. 2019 Jul 22;15(7):e1008251. doi: 10.1371/journal.pgen.1008251 (PMC6690552; doi:10.1371/journal.pgen.1008251)
Supplement: S3 Table — Showns are the numbers of amplified Y genes identified, for different cut-offs of male/female coverage ratio (M/F from 2.5 to 10). (PDF) [file pgen.1008251.s012.pdf]

Table S3. Amplified Y genes vs. M/F cutoffs. Showns are the number of amplified Y genes identified, for different cut-off's of male/female coverage ratio (M/F from 2.5 to 10).

cutoff	#genes
M/F>=2.5	2000
M/F>=3	954
M/F>=3.5	542
M/F>=4	346
M/F>=4.5	258
M/F>=5	205
M/F>=5.5	178
M/F>=6	154
M/F>=6.5	133
M/F>=7	117
M/F>=7.5	104
M/F>=8	95
M/F>=8.5	84
M/F>=9	75
M/F>=9.5	69
M/F>=10	64
