# Supplementary material for: Recurrent gene co-amplification on Drosophila X and Y chromosomes
Source: PLoS Genet. 2019 Jul 22;15(7):e1008251. doi: 10.1371/journal.pgen.1008251 (PMC6690552; doi:10.1371/journal.pgen.1008251)
Supplement: S4 Table — Shown are the orthologous location of multi-copy Y genes in D. melanogaster, and their inferred molecular function and gene expression pattern in D. melanogaster (data from flybase.org). (PDF) [file pgen.1008251.s013.pdf]

Table S4. Multi-copy Y-linked genes across Drosophila species. Shown are the orthologous location of multi-copy Y genes in *D. melanogaster*, and their inferred molecular function and gene expression pattern in *D. melanogaster* (data from flybase.org).

Species	Chromosome	Peptide ID	Gene ID	InterPro Domain	Molecular Function	Testes_expression	Highest_Expression_Tissue	Highest_Expression_Development	Name	Gene Snapshot
D. albomicans	3L	FBpp0091021	FBgn0053725	Protein of unknown function DUF1091	NA	NA	NA	NA	NA	NA
D. americana	2L	FBpp0080666	FBgn0032715	Thiolase	transferase activity, transferring acyl groups other than amino-acyl groups	Y	Digestive System	Larva	NA	NA
D. athabasca	2R	FBpp0071874	FBgn0045483	7TM chemoreceptor	taste receptor activity	NA	NA	NA	Gr59a	NA
D. athabasca	2R	FBpp0085702	FBgn0034429	GNAT domain	N-acetyltransferase activity	N	Digestive System	Larva	NA	NA
D. athabasca	2R	FBpp0085908	FBgn0034328	Immune-induced protein Dim	NA	N	Female Head	Adult Male	IM23	NA
D. athabasca	3L	FBpp0088483	FBgn0036640	Leucine-rich repeat	May be involved in the export of mRNA from the nucleus to the cytoplasm	Y	Ovary	Embryo,Adult Female	nxf2	NA
D. lummei	X	FBpp0070717	FBgn0011761	Thioredoxin	female meiotic nuclear division, disulfide oxidoreductase activity	Y	Ovary	Embryo, Adult Female	dhd	Deadhead is a thioredoxin-like protein necessary for the initiation of embryonic development.
D. melanica	3L	FBpp0075822	FBgn0028668	V-ATPase proteolipid subunit	proton-transporting ATPase activity, rotational mechanism	Y	Testes	Adult Male	Vha16-2	NA
D. melanica	3L	FBpp0088546	FBgn0010424	EF-hand domain	calcium ion binding	Y	Carcass of larva	Embryo,Larva,Pupae	TpnC73F	NA
D. melanica	3L	FBpp0091046	FBgn0053796	Protein of unknown function DUF1091	NA	NA	NA	NA	NA	NA
D. miranda	2R	FBpp0071501	FBgn0026582	High mobility group box domain	nucleus	Y	Ovary	Embryo	Hmg-2	NA
D. miranda	2R	FBpp0072323	FBgn0060296	Ankyrin repeat	calcium channel activity, male courtship behavior	Y	Carcass of larva	Embryo,Larva,Pupae	pain	NA
D. miranda	3L	FBpp0072616	FBgn0004378	Kinesin motor domain; Kinesin-associated microtubule-binding domain	ATP-dependent microtubule motor activity	Y	Ovary	Embryo	Klp61F	Kinesin-like protein at 61F (Klp61F) is a member of the kinesin-5 family of cytoskeletal motor proteins. Klp61F allows the crosslinking and sliding apart of adjacent microtubules. This 'sliding filament mechanism' is critical for many aspect of mitosis and chromosome segregation.
D. miranda	2R	FBpp0085582	FBgn0034491	Hormone-sensitive lipase, N-terminal	lipase activity	Y	Ovary	Embryo	Hsl	Hormone-sensitive lipase is involved in lipid storage
D. miranda	2R	FBpp0085619	FBgn0005655	Proliferating cell nuclear antigen, PCNA	DNA binding, mitotic spindle organization	Y	Ovary	Embryo,Adult Female	PCNA	NA
D. miranda	2R	FBpp0085706	FBgn0034435	NA	spindle assembly involved in male meiosis I	Y	Testes,Imaginal Disc	Adult Male	fest	NA
D. miranda	2R	FBpp0086004	FBgn0003701	NA	protein binding,mitotic sister chromatid segregation	Y	Ovary	Embryo	thr	Three rows (Thr) together with Sse forms the endoprotease separase complex, which cleaves a subunit of the cohesin complex, thereby allowing separation of the sister chromatids.
D. miranda	2R	FBpp0086750	FBgn0033860	Peptidase M17, leucine aminopeptidase/peptidase B	aminopeptidase activity	Y	Testes	Adult Male	S-Lap5	NA
D. miranda	2R	FBpp0086788	FBgn0033845	SAPAP family	microtubule binding,chromosome segregation	Y	Testes,Ovary	Embryo	mars	NA
D. miranda	2R	FBpp0086910	FBgn0033788	Transcription activator MBF2	NA	Y	Digestive System	Larva	NA	NA
D. miranda	2R	FBpp0087756	FBgn0033354	Fanconi anemia group I protein	DNA polymerase binding,mitotic G2 DNA damage checkpoint	Y	Ovary	Embryo	FANCI	NA
D. miranda	2R	FBpp0087986	FBgn0261385	Pleckstrin homology domain; Anillin homology domain	actin binding,male meiosis cytokinesis	Y	Ovary	Embryo	scra	Scraps is a homolog of anilin, a conserved pleckstrin homology domain (PLEKH) containing protein that binds actin, nonmuscle myosin II and microtubules. It stabilizes the contractile ring and is required for completion of cytokinesis

D. miranda	2R	FBpp0088003	FBgn0033216	Diacylglycerol acyltransferase	transferase activity	Y	Digestive System	Embryo,Adult Male	NA	NA
D. miranda	X	FBpp0290242	FBgn0052666	Protein kinase domain	protein serine/threonine kinase activity	Y	Ovary, Salivary glad of prepupae	Embryo,Pupae,Adult Female	Drak	Death-associated protein kinase related (Drak) is a protein serine/threonine kinase belonging to the Death-associated protein kinase family. Drak promotes phosphorylation of sqh at sites known to stimulate actomyosin contractility. Its main characterized function is to shape epithelial tissues during development.
D. miranda	2R	FBpp0291585	FBgn0054045	NA	NA	NA	NA	NA	NA	NA
D. nannoptera	X	FBpp0077024	FBgn0031103	Protein of unknown function DUF4791	NA	Y	Testes	Adult Male	NA	NA
D. nigromelanica	2R	FBpp0085858	FBgn0063491	Glutathione S-transferase, N-terminal	glutathione transferase activity	Y	Head	Larva,Adult Male	GstE9	NA
D. nigromelanica	2R	FBpp0086954	FBgn0011604	ISWI family	DNA-dependent ATPase activity; nucleosome-dependent ATPase activity; protein binding; transcription factor binding; sperm chromatin condensation; spermatogenesis	Y	Ovary, Imaginal Disc and CNS of larva	Embryo	Iswi	NA
D. pseudoobscura	3L	FBpp0076362	FBgn0035915	Peptidase M17, leucine aminopeptidase/peptidase B	aminopeptidase activity	Y	Testes	Adult Male	S-Lap1	NA
D. pseudoobscura	3L	FBpp0076363	FBgn0052351	Peptidase M17, leucine aminopeptidase/peptidase B	aminopeptidase activity	Y	Testes	Adult Male	S-Lap2	NA
D. pseudoobscura	3L	FBpp0076372	FBgn0035916	Rab-GTPase-TBC domain	GTPase activator activity	Y	Ovary	Embryo	GAPsec	NA
D. pseudoobscura	3L	FBpp0112062	FBgn0035690	Zinc finger, AD-type; Zinc finger, RING/FYVE/PHD-type; Zinc finger C2H2-type	transcription factor activity, sequence-specific DNA binding	Y	Ovary	Embryo	NA	NA
D. robusta	3L	FBpp0072987	FBgn0010317	Cyclin-like	cyclin-dependent protein serine/threonine kinase regulator activity; mitotic cell cycle	Y	Ovary	Embryo, Adult Female	Cyclin J	NA
D. robusta	3L	FBpp0078102	FBgn0046301	Leucine-rich repeat domain	ubiquitin-protein transferase activity	Y	Testes	Embryo	NA	NA
D. robusta	3L	FBpp0312385	FBgn0053795	Protein of unknown function DUF1091	NA	NA	NA	NA	NA	NA
