# Supplementary material for: Recurrent gene co-amplification on Drosophila X and Y chromosomes
Source: PLoS Genet. 2019 Jul 22;15(7):e1008251. doi: 10.1371/journal.pgen.1008251 (PMC6690552; doi:10.1371/journal.pgen.1008251)
Supplement: S5 Table — (PDF) [file pgen.1008251.s014.pdf]

Table S5. Inferred copy numbers for co-amplified X and Y genes

Species	Gene	X copies in assembly	Inferred Y Copy Number
D. albomicans	*FBgn0053725*	2	16
D. americana	*FBgn0032715*	3	13
D. athabasca	*FBgn0034328*	2	9
D. athabasca	*FBgn0034429*	4	16
D. athabasca	*FBgn0036640*	2	12
D. athabasca	*FBgn0045483*	3	16
D. lummei	*dhd*	2	10
D. melanica	*FBgn0010424*	2	9
D. melanica	*FBgn0028668*	2	8
D. melanica	*FBgn0053796*	2	8
D. miranda	*Drak*	2	232
D. miranda	*FBgn0026582*	2	39
D. miranda	*FBgn0033216*	2	10
D. miranda	*FBgn0033354*	3	15
D. miranda	*FBgn0033788*	2	8
D. miranda	*FBgn0034491*	2	8
D. miranda	*FBgn0054045*	2	14
D. miranda	*FBgn0060296*	2	186
D. miranda	*fest*	4	147
D. miranda	*Klp61F*	3	18
D. miranda	*mars*	2	32
D. miranda	*PCNA*	2	8
D. miranda	*S-Lap5*	3	82
D. miranda	*scra*	4	289
D. miranda	*thr*	2	35
D. nannoptera	*FBgn0031103*	2	158
D. nigromelanica	*FBgn0063491*	3	33
D. nigromelanica	*Iswi*	2	8
D. pseudoobscura	*FBgn0035690*	3	19
D. pseudoobscura	*GAPsec*	2	68
D. pseudoobscura	*S-Lap1*	2	61
D. pseudoobscura	*S-Lap2*	2	159
D. robusta	*FBgn0010317*	2	8
D. robusta	*FBgn0046301*	2	297
D. robusta	*FBgn0053795*	2	9
