# Supplementary material for: Recurrent gene co-amplification on Drosophila X and Y chromosomes
Source: PLoS Genet. 2019 Jul 22;15(7):e1008251. doi: 10.1371/journal.pgen.1008251 (PMC6690552; doi:10.1371/journal.pgen.1008251)
Supplement: S6 Table — Shown are inferred copy numbers of co-amplified X and Y genes, based on our analysis, and compared to high-quality genome assemblies from D. miranda and D. pseudoobscura. (PDF) [file pgen.1008251.s015.pdf]

Table S6. Comparison of inferred copy numbers of co-amplified X and Y genes. Shown are inferred copy numbers of co-amplified X and Y genes, based on our analysis, and compared to high-quality genome assemblies from *D. miranda* and *D. pseudoobscura*.

				Y copies long read assembly					X copies long read assembly				
Genes	*D. mel* homolog	Y copies M/F cov	X copies assembly	Y total >25%	>0.9	0.9-0.5	0.5-0.25	<0.25	X total >25%	>0.9	0.9-0.5	0.5-0.25	<0.25
D. miranda													
FBgn0064414	*thr*	35	2	28	20	5	3	8	1	1	0	0	0
FBgn0070787	*FBgn0034491*	8	2	4	3	1	0	0	1	1	0	0	0
FBgn0072252	*FBgn0033788*	8	2	2	0	2	0	0	2	2	0	0	0
FBgn0074335	*mars*	32	2	48	36	10	2	1	6	2	2	2	0
FBgn0074933	*FBgn0033860*	82	3	77	60	15	2	3	2	1	1	0	2
FBgn0075168	*FBgn0033216*	10	2	3	1	0	2	0	3	1	0	2	0
FBgn0075258	*scra*	289	4	9	6	1	2	2	2	1	0	1	2
FBgn0077065	*Drak*	232	2	49	0	0	0	60; 49>1kb	1	1	0	0	44
FBgn0081586	*Klp61F*	18	3	27	0	23	4	44	4	4	0	0	1
FBgn0081588	*PCNA*	8	2	4	4	0	0	0	1	1	0	0	0
FBgn0081758	*FBgn0026582*	39	2	69	1	0	68	4	1	1	0	0	0
FBgn0082139	*fest*	147	4	145	96	43	6	10	5	3	2	0	0
FBgn0245922	*FBgn0060296*	186	2	181	0	156	25	23	1	1	0	0	0
FBgn0246178	*FBgn0054045*	14	2	2	0	2	0	60	1	1	0	0	0
FBgn0246244	*FBgn0033354*	15	3	9	0	8	1	3	14	1	0	11	2; 2>1kb
D. pseudoobscura													
FBgn0079543	*S-Lap1*	159	2	127	21	62	18	26	2	1	0	1	0
FBgn0250028	*GAPsec*	68	2	95	6	22	22	45	2	1	0	1	0
FBgn0070269	*FBgn0035690*	19	3	4	3	1	0	0	3	1	0	2	0
