# Supplementary material for: Recurrent gene co-amplification on Drosophila X and Y chromosomes
Source: PLoS Genet. 2019 Jul 22;15(7):e1008251. doi: 10.1371/journal.pgen.1008251 (PMC6690552; doi:10.1371/journal.pgen.1008251)
Supplement: S7 Table — (PDF) [file pgen.1008251.s016.pdf]

Table S7. GO functions of multi-copy Y genes, and genes co-amplified on the X and Y.

GO function	Description	P-value
**co-amplified X/Y genes**		
GO:0035041	sperm chromatin decondensation	2.61E-04
GO:0007051	spindle organization	4.86E-04
GO:0022402	cell cycle process	6.44E-04
GO:0007052	mitotic spindle organization	9.68E-04
**multi-copy Y genes**		
GO:0006518	peptide metabolic process	2.37E-09
GO:0006412	translation	4.79E-08
GO:0043043	peptide biosynthetic process	9.32E-08
GO:0043604	amide biosynthetic process	6.40E-07
GO:0043603	cellular amide metabolic process	9.34E-07
GO:0032543	mitochondrial translation	1.25E-05
GO:0044271	cellular nitrogen compound biosynthetic process	2.03E-05
GO:0015985	energy coupled proton transport	2.93E-05
GO:0015986	ATP synthesis coupled proton transport	2.93E-05
GO:0034645	cellular macromolecule biosynthetic process	5.95E-05
GO:0009144	purine nucleoside triphosphate metabolic process	6.18E-05
GO:0034622	cellular protein-containing complex assembly	6.21E-05
GO:0009141	nucleoside triphosphate metabolic process	6.98E-05
GO:0046034	ATP metabolic process	9.17E-05
GO:0009123	nucleoside monophosphate metabolic process	9.36E-05
GO:0065003	protein-containing complex assembly	1.03E-04
GO:0022900	electron transport chain	1.14E-04
GO:0009059	macromolecule biosynthetic process	1.40E-04
GO:0009205	purine ribonucleoside triphosphate metabolic process	1.66E-04
GO:0002181	cytoplasmic translation	1.75E-04
GO:0022904	respiratory electron transport chain	1.92E-04
GO:1901566	organonitrogen compound biosynthetic process	1.95E-04
GO:0009199	ribonucleoside triphosphate metabolic process	2.05E-04
GO:1990542	mitochondrial transmembrane transport	2.30E-04
GO:1902600	proton transmembrane transport	2.84E-04
GO:0009126	purine nucleoside monophosphate metabolic process	3.74E-04
GO:0009167	purine ribonucleoside monophosphate metabolic process	3.74E-04
GO:0009161	ribonucleoside monophosphate metabolic process	3.89E-04
GO:0006091	generation of precursor metabolites and energy	4.87E-04
GO:0043933	protein-containing complex subunit organization	5.16E-04
GO:0045333	cellular respiration	5.48E-04
GO:0009124	nucleoside monophosphate biosynthetic process	6.31E-04
GO:0006749	glutathione metabolic process	7.63E-04
