# Supplementary material for: Recurrent gene co-amplification on Drosophila X and Y chromosomes
Source: PLoS Genet. 2019 Jul 22;15(7):e1008251. doi: 10.1371/journal.pgen.1008251 (PMC6690552; doi:10.1371/journal.pgen.1008251)
Supplement: S8 Table — (PDF) [file pgen.1008251.s017.pdf]

Table S8. Mapping of total RNA and short RNA for X- and Y-linked copies of *GAPsec* and *S-Lap1*

		total RNA*		short RNA*	
gene	chromosome	sense	anti-sense	sense	anti-sense
GAPsec	X	501	5	38	12
*GAPsec*_dup	X	60	68	37	7
*GAPsec*_consensus	Y	20	26	46	47
S-Lap1	X	39564	4	360	94
*S-Lap1*_dup	X	168	418	569	5465
*S-Lap1*_consensus	Y	27033	21	3862	858

*Reads per kb
Alignments >= MQ 20
