# Supplementary material for: Recurrent gene co-amplification on Drosophila X and Y chromosomes
Source: PLoS Genet. 2019 Jul 22;15(7):e1008251. doi: 10.1371/journal.pgen.1008251 (PMC6690552; doi:10.1371/journal.pgen.1008251)
Supplement: S9 Table — Shown is the expression of the sense and antisense transcript, and expression of small RNA, and the location of paralogs in the D. pseudoobscura genome. Gene expression data are given in S5 Data. (PDF) [file pgen.1008251.s018.pdf]

**Table S9. Genes showing antisense transcription and targeting by short RNAs in *D. pseudoobscura* testis.** Shown is the expression of the sense and antisense transcript, and expression of small RNA, and the location of paralogs in the *D. pseudoobscura* genome.

| *D. pse* Gene ID | *D. pse* Transcript | Expression (RPM) Sense transcript | Antisense transcript | Small RNA | *D. mel* homolog | *D. pseudoobscura* duplicates location |
|---|---|---|---|---|---|---|
| *GA25902* | FBtr0281975 | 8.5 | 120.4 | 320.6 | *CG15800* | 4_group3[1 copy]; XL_group1a[1 copy] |
| *GA28949* | FBtr0370834 | 30.3 | 43.8 | 420.8 | *ACX* | 4_group4[2 copies] |
| *GA27727* | FBtr0377521 | 12.4 | 36.7 | 386.6 | *Svil* | 4_group2[2 copies]; XR_group6[1 copy]; Unknown_singleton_1069[2 copies] |
| *GA31882* | FBtr0376742 | 7.8 | 17.8 | 450.9 | *none* | 4_group3[1 copy], Unknown_singleton_1325[1 copy]; Unknown_group_211[ 1 copy]; Unknown_group_669[3 copies]; *Y[2 copies: Unknown_singleton_2735, Unknown_singleton_1347] |
| *GA32779* | FBtr0372083 | 8.4 | 13.9 | 288.0 | *none* | 2[1 copy], XR_group8[5 copies], Unknown_singleton_374[1 copy] |
| *GA31908* | FBtr0372976 | 4.4 | 3.4 | 741.7 | *none* | XL_group1e[1 copy]; *Y[4 copies: Unknown_singleton_18[2 copies], Unknown_singleton_1146, Unknown_singleton_1161]; Unknown_group_669[5 copies], Unknown_singleton_1699[1 copy] |

* For some “Unknown” genes, we could infer their Y-linked location based on male-specific coverage.
